# Establishing multi-perspective instruments in early education during COVID-19: measuring the implementation of protective measures and the subjective level of information about pandemic-related regulations

**DOI:** 10.1186/s42409-022-00033-2

**Published:** 2022-05-12

**Authors:** Johannes Wieschke, Diana D. Schacht, Florian Spensberger, Nicole Klinkhammer, Mariana Grgic

**Affiliations:** 1 German Youth Institute, Deutsches Jugendinstitut eV, Munich, Germany; 2 Catholic University of Applied Sciences Munich/Catholic University Eichstätt-Ingolstadt, Munich, Germany

## Abstract

This article describes the context, development, objectives, and content of three instruments. They stem from two questionnaires, used in the ERiK-Surveys 2020 and the Corona-KiTa-Study (CKS), two multi-perspective surveys which were developed by the German Youth Institute, to measure quality as well as challenges and solutions of the Corona pandemic in early childhood education and care (ECEC). The three instruments focus on (1) childcare center directors’ subjective level of information about pandemic-related regulations in the ERiK questionnaire and the extent of implementation of (2) hygiene and (3) protective measures in ECEC in the CKS questionnaire. First analyses suggest good performance and quality of the instruments. Further analyses (e.g., regarding validity and reliability) will be carried out. The instruments seem to be promising for future research, for example regarding medical questions in the field of ECEC.

**Supplementary Information:**

The online version contains supplementary material available at 10.1186/s42409-022-00033-2.

## Background

In 2019, the law on improving quality and participation in early childhood education and care^1^ (ECEC) came into effect in Germany with the aim of improving supply and quality of childcare. Subsequently, the German Youth Institute (DJI) launched the research-project *Indicator-based monitoring of structural quality in the German early childhood education and care system* (ERiK study). The aim of the project is to develop an indicator-based^2^ monitoring approach that captures the situation and changes of quality within the ECEC system, considering the view of different stakeholders in the field (cf. Klinkhammer et al., 2021). Respective monitoring questionnaires were developed. To address the outbreak of the COVID-19 pandemic at the beginning of 2020, these questionnaires were enhanced with instruments related to quality in ECEC during the pandemic (e.g., regulation of access to ECEC).

To capture pandemic-related challenges and solutions (e.g., regarding day care, cooperation, well-being, hygiene, and protective measures) from different stakeholders in ECEC, the Corona-KiTa-Study (CKS), a cooperation project between the DJI and the Robert Koch Institute (RKI), was launched in May 2020. Two of six CKS surveys used sub-samples from the samples (center directors, childminders) of the ERiK study. This way, the CKS directors’ instrument presented here is connected to the ERiK study and can also make use of the information gathered there. Additionally, two CKS samples of parents and pedagogical staff were recruited with the help of the already interviewed directors, making further connections possible.

The ERiK-surveys 2020^3^ were partly conducted in the first wave of the pandemic (April–September 2020), and parts of the CKS were conducted during the second and third wave of the pandemic and beyond (October 2020–July 2021) (see Additional file 1) Table 1 gives an overview of the six different questionnaires and samples (i.e., directors of and pedagogical staff in ECEC centers, parents of children in day-care^4^, childminders, providers of childcare and youth welfare offices) of the ERiK- and Corona-KiTa-Study, containing the discussed pandemic instruments.

This article focuses on three instruments, i.e. three sets of items from the two studies (ERiK and CKS). They are part of the respective ECEC center directors’ questionnaires of each study, which are exemplary for the set of multi-perspective questionnaires on quality as well as on challenges and solutions in ECEC settings during the COVID-19 pandemic. The context, development, objectives and content of the three instruments are described, one concerning the directors’ subjective level of information (ERiK – see Additional file 2, in particular the first two items in question 4, which will be presented descriptively later on) and the other ones regarding hygiene and protective measures in ECEC centers (CKS) (see Additional file 3, questions 19 and 20). Furthermore, we assess their related data quality and provide further insights on the strengths and weaknesses of the questionnaires and instruments.

## Instrument context and description

With the worldwide outbreak of the COVID-19 pandemic, the German ECEC system was faced with new challenges on various levels of responsibility—on the level of federal states, on the level of youth welfare offices and providers of ECEC settings as well as on the level of directors of day care centers. Hence, the development of our survey instruments was guided by the inclusion of different perspectives at both individual and institutional level.

For example, the regulation of access for children to the ECEC system during reduced supply (cf., Meiner-Teubner, 2020) as well as the implementation of hygiene and protective measures in ECEC settings belonged to the most important, but also challenging issues. Directors of ECEC centers needed information regarding these issues from different stakeholders to face respective challenges appropriately. To address the latter, an instrument was developed to measure the ECEC center director’s subjective level of information on pandemic-related regulations provided by relevant stakeholders (ERiK instrument, see Additional file 2). Furthermore, two instruments were developed to measure the extent to which hygiene and protective measures were implemented in ECEC centers (CKS instrument, see Additional file 3).

### ERiK instrument: ECEC center directors’ subjective level of information regarding pandemic-related regulations

The ERiK study was scheduled to go into field in March 2020 with five questionnaires developed on the basis of previous ECEC surveys covering the ten qualitative fields of action and measures to ease burden of fees on families defined in the “Further Development of Quality and the Improvement of Participation in Day-Care Facilities and in Child Day-Care” Act (Bundesgesetzblatt, 2018). However, the planned field start was postponed from March 2020 to April resp. May 2020 with the outbreak of the pandemic. The delay was used to adapt the survey programme to the new situation during the first wave of the pandemic. Thus, newly developed instruments explicitly addressing specifics on the changed Corona situation in the field of ECEC have been supplemented additionally to each questionnaire. These add-ons included instruments on more obvious ECEC problems (e.g., emergency care) as well as the potentially less obvious problems (e.g., information deficits of pedagogical staff regarding necessary health measures).

The add-on for directors contained 5 questions covering (1) the regulation of access to ECEC, (2) the capacity of places, (3) the cooperation of local stakeholders, (4) the subjective level of information regarding pandemic-related regulations such as protection of ECEC staff, protective measures for children, regulations for the deployment of staff, and (5) local agreements for organizing care for children whose parents work in profession within the health or public system was also included (1 question, 6 items).^5^ Directors were asked to answer the latter two questions with 8 items on a 6-point Likert Scale (1 “very poor”–6 “very good”). These issues were particularly relevant for political stakeholders in the early stages of the pandemic, since there was no experience on how to efficiently govern the ECEC system in such an exceptional situation.

### CKS instrument: implementation of hygiene and protective measures in ECEC centers

The questionnaires of the various CKS surveys focus on pandemic-related challenges and solutions in ECEC such as the demand for ECEC services, how this demand has changed during the pandemic or the amount of day care that is available against the background of COVID-19-related health guidelines. An extensive part of the questionnaires also addresses hygiene and protective measures that can be observed by parents, children and ECEC center directors.

Directors interviewed here are a sub-sample of the directors already interviewed in the ERiK study, so that the information gathered there is also available for the CKS. The CKS instrument of the directors’ survey was developed to measure the extent to which hygiene and protective measures were implemented in ECEC centers. The directors’ questionnaire comprises 28 questions with 279 items and covers the implementation of recommended measures, detailed information about measures in specific situations (e.g., lunch situation, contacts with parents, using of sanitary or outdoor area) and difficulties regarding the spatial situation or implementation. Eleven questions and 73 items are directly COVID-related. For the development of this new set of items, a preliminary study was conducted, based on qualitative interviews with 83 directors (cf. Autorengruppe Corona-KiTa-Studie, 2020). Supplementary research was carried out on recommended measures for ECEC centers at the level of federal states. Items were also reviewed by researchers from the RKI.

Due to the large scope of the questionnaire, this paper is limited to the presentation and discussion of the two instruments regarding basic hygiene and protective measures not applicable to specific situations (2 questions—numbers 19 and 20—with 16 and 7 items, respectively). Other items deal not only with different topics related to this (e.g., number of infections), but also with not directly COVID-related issues such as the situation before the pandemic. The items presented here relate to the implementation of 16 measures by staff, parents or children and to the situations in which pedagogical staff is wearing face masks. Directors are asked to rate the extent to which certain protective and hygiene measures were implemented in their ECEC center on a 5-point Likert scale (1 “very poor”–5 “very good”). Items of the first instrument can be clustered into four different categories:

1. Reduction of contacts (4 items, e.g., group separation inside the ECEC center, fixed staff assignment to groups),
2. Reduction of aerosols transmission (6 items, e.g., wearing a face mask, “staff keep distance from each other”),
3. Protection of contact transmission (3 items, e.g., shaking hands with children on a regular basis, disinfection),
4. Handling (the lack of) symptoms (5 items, e.g., “daily temperature measurement”, testing staff).

The second instrument goes into further detail regarding the frequency of mask-wearing among staff in different situations (e.g., contact with parents or other staff) (scale from 0 “never” to 5 “always”). Due to the multi-perspective and longitudinal nature of most parts of the study, many of the measurements can also be observed at different points in time. This enables researchers to investigate changes in variable values and to examine these changes for example in the context of local rates of infections or in the context of answers given by other respondents connected to the same institution, widening the scope of possible studies.

## Aspects of data quality

Regarding the development of the three instruments discussed here, we found little orientation in previous studies due to the novelty of the pandemic-related topics. Hence, the instruments had to be developed largely from scratch. Even though deeper analyses regarding data quality were carried out across the multi-perspective set of questionnaires in the ERiK-study, this was only possible to some extent for the three ERiK-/CKS-instruments due to time-constraints before the field launches. First analyses indicate that reliability and validity of the described three instruments are adequate, but further exploration will be carried out.

In the ERiK study, data quality was assessed based on content validity (e.g., Sireci, 1998), usefulness (e.g., Benova, 2020), distributions of variables, missingness patterns and average response time. In a first step, the distributions and missingness patterns of the related variables have been critically examined, e.g., whether distributions are skewed, the number of missings and whether these missings occurred particularly frequently in some populations. We considered, for example, an item-non response of 7% on the two items on the subjective level of directors’ informedness regarding pandemic-related regulations such as the protection of ECEC staff and protective measures for children as rather high. However, the probability to not response on both items did not statistically significantly vary along some core demographic characteristics of directors (gender, age, education at alpha = 0.05).

This first evaluation process allowed for a quick evaluation of the newly generated instrument. At the same time, the average processing time of the overall web questionnaires was rather high (between 35 min for pedagogical staff, about 55 min for directors^6^ and 105 minutes for youth welfare offices, Klinkhammer et al., in prep). In survey methodology, questionnaires that take more than 30 min to complete are often considered “unreasonable,” and too long questionnaires can be one of the main sources of erroneous responses (Tourangeau et al., 2009). Hence, these durations may have influenced the sample composition and the response behavior. Indeed, in two additional non-response surveys of providers and directors, evidence shows that largely time restrictions led to a non-response, e.g., 17% of the directors considered the questionnaire too long and therefore did not participate in the ERiK-Surveys 2020 (cf. Schacht et al., in prep). These steps provided the basis for an extensive reduction of the questions for a renewed cross-sectional survey in 2022. The directors’ questionnaire was shortened by 13 questions reducing the overall number of questions to 90. Particularly relevant for the reductions was the extent to which the items were also classified as still useful. Usefulness was assumed if the items were analyzed and presented in a research report for the project and also if external specialists considered the questions relevant for 2022. For this purpose, all questions were discussed together in three sessions with regard to their usefulness. Questions omitted include those from the ERiK instrument presented here, as it was assumed that the ERiK instrument would capture specific pandemic-related information that could not be collected in 2022.

For the ERiK overall questionnaire, descriptive statistics based on the net sample of the ERiK-Surveys 2020 were compared with information from the National Child and Youth Welfare Statistics, which contains information about the children attending ECEC and the staff working in the field (cf. Statistisches Bundesamt, 2020) (external validity). For the survey of directors, slight variations in these descriptive statistics were found. Directors with higher volumes of employment or with a university degree relevant to their work compared to other degrees were overrepresented (compared to the KJH; based on a chi-square goodness of fit test being statistically significant at alpha = 0.05). This marginal bias of the two socio-demographic variables studied was compensated for by a corresponding weighting procedure (cf. Schacht et al. in prep.).^7^ With regard to the ERiK instrument presented here, however, such an external validity comparison with the National Child and Youth Welfare Statistics was not possible, as the latter do not contain any corresponding information on, e.g., directors’ subjective level of informedness on protection of ECEC staff and protective measures for children.

**Fig. 1:**
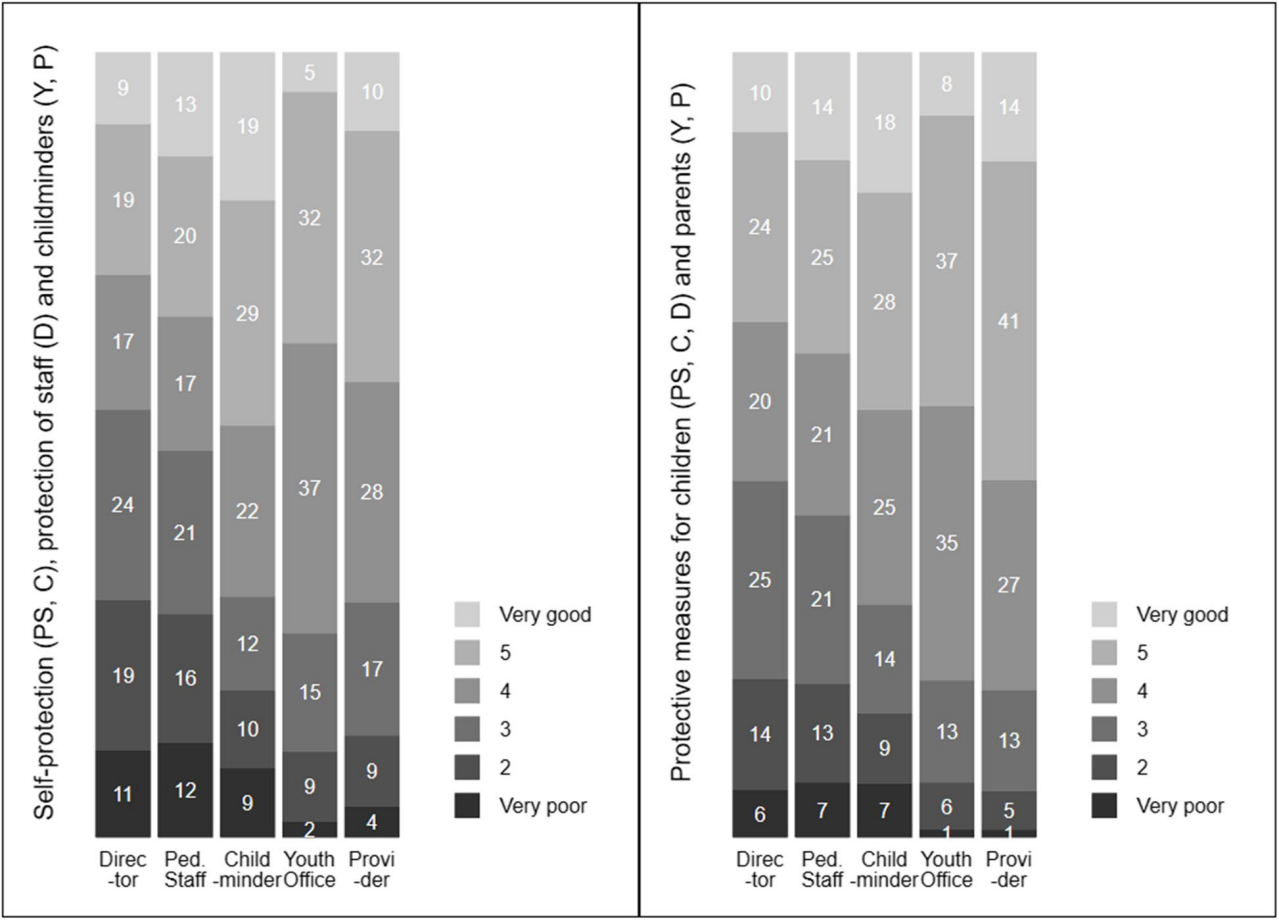
Directors’ subjective level of informedness on protection of ECEC staff and protective measures for children compared to information given by the other four ERiK target populations. Legend: as reported by directors (D), pedagogical staff (PS), childminders (C), youth offices (Y) and providers (P), ERiK-Surveys 2020, weighted results

Nonetheless, comparing directors’ subjective level of informedness on protection of ECEC staff and protective measures for children with the views of other stakeholders captured in the ERiK-Surveys 2020 gives a sense of the data quality achieved with these two items. As can be seen in Fig. 1, directors felt subjectively less well informed compared to other ECEC stakeholders regarding both items. The ERiK Team and experts from the field would have expected that agencies and youth welfare offices would have felt particularly well informed, since they are in particularly close contact with other relevant stakeholders such as the state health authorities. It also seems plausible that, on average across all stakeholders, information on protective measures for children (resp. parents) was better than that on self-protection, protection of staff and childminders (depending on the target population). In this respect, the patterns presented here appear extremely realistic for the ERiK team and experts that discussed the results with us. The preliminary results confirm the data quality achieved with these two new items included in the ERiK instrument.^8^

Regarding data quality of the CKS, only limited comparisons with results of official statistics were possible due to the new development of pandemic-related items in the study. However, since the sample consisted of ERiK participants, CKS respondents can be compared to the former group in order to determine whether non-response was random. Results of a chi-square goodness of fit test show that the state of Berlin (3.8% in the original sample versus 2.9% among respondents) and municipalities with at least 500,000 inhabitants (12.1 versus 10.6%) are slightly underrepresented, and those with less than 2000 inhabitants overrepresented (5.4 versus 9.9%) compared to the ERiK data. The average response time for the CKS directors’ questionnaire was about 35 minutes (SD = 13.72), which also exceeds the suggested 30-min threshold.

Although only certain items are analysed here, comparisons of the director’s data are possible on multiple levels: with ERiK data, as described above, with external data, with the directors’ data itself, but from a different measurement point, or with other data from the CKS that are connected to the directors’ questionnaire.

To catch changes over time—both within and between centers or persons—, the study uses multiple measurement points in most surveys. In case of the directors’ survey, four groups of directors (with differing starting dates) were each interviewed twice, with about four months between the two measurement points (questionnaires did not differ between groups, but between measurement points). This is especially helpful against the background of a dynamic infection process in Germany during this study, because specific measures and events in ECEC centers depend strongly on the regional infection situation and data can then also be contrasted with changes in infection numbers.

**Fig. 2:**
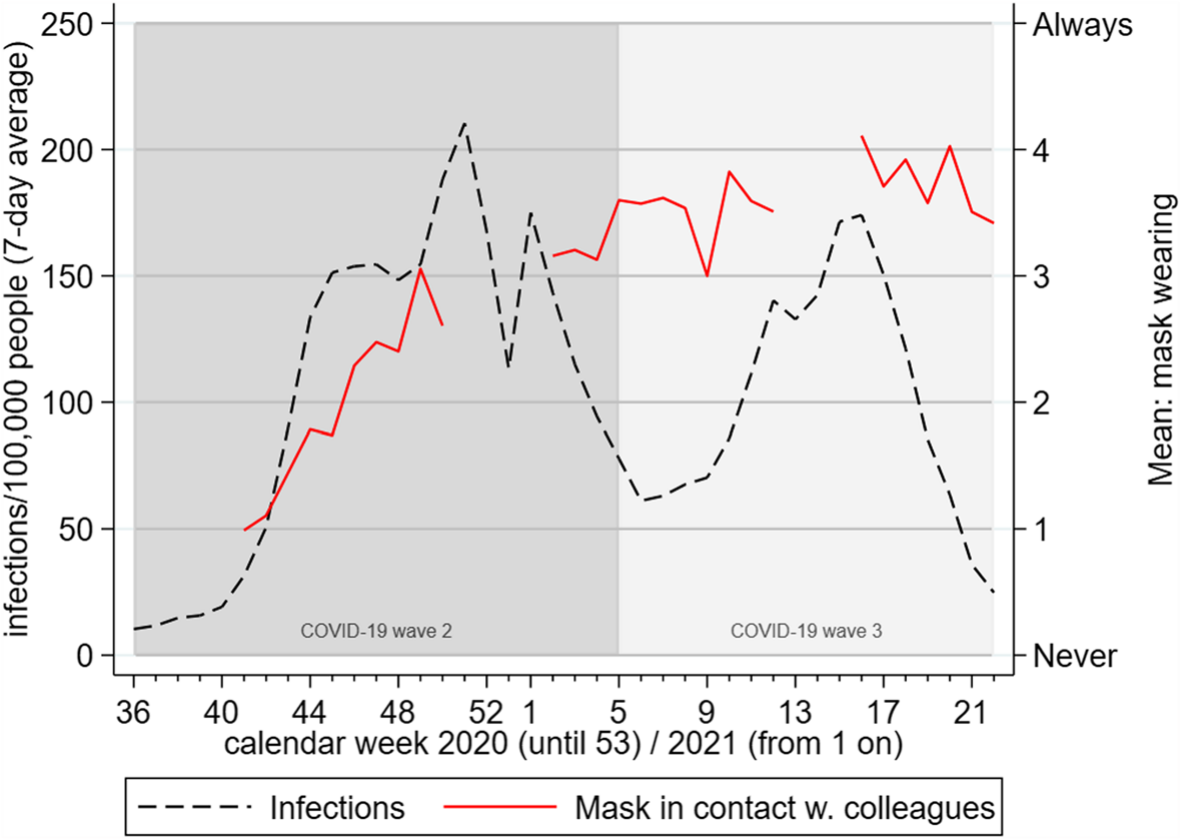
Weekly COVID-19 infection rates and prevalence of mask-wearing of pedagogical staff in interactions with colleagues. Legend: as reported by directors in the respective ECEC centers, CKS-Survey 2020

The CKS-instruments contain 16 items with a 5-point Likert scale and 7 items with a 6-point Likert scale, respectively, for a wide variety of measures, enabling respondents to indicate even small changes for example in the prevalence of mask-wearing or of group separations in ECEC settings. Together with data of the COVID-19 cases in the population, this makes it possible to compare behavioral and infection trends, as can be seen in Fig. 2. The lines clearly show that during the “second wave” in the autumn and winter of 2020/2021, infection rates as well as the likelihood of pedagogical staff wearing facemasks changed significantly. After that, infection rates again decreased, while the prevalence of mask-wearing remained largely unchanged, possibly due to habituation effects or due to mask mandates staying in place. Analyses such as this one can serve as external validity tests, when data from the survey is merged with data from other sources, even though this figure can only show correlation and no causal effect.

**Fig. 3:**
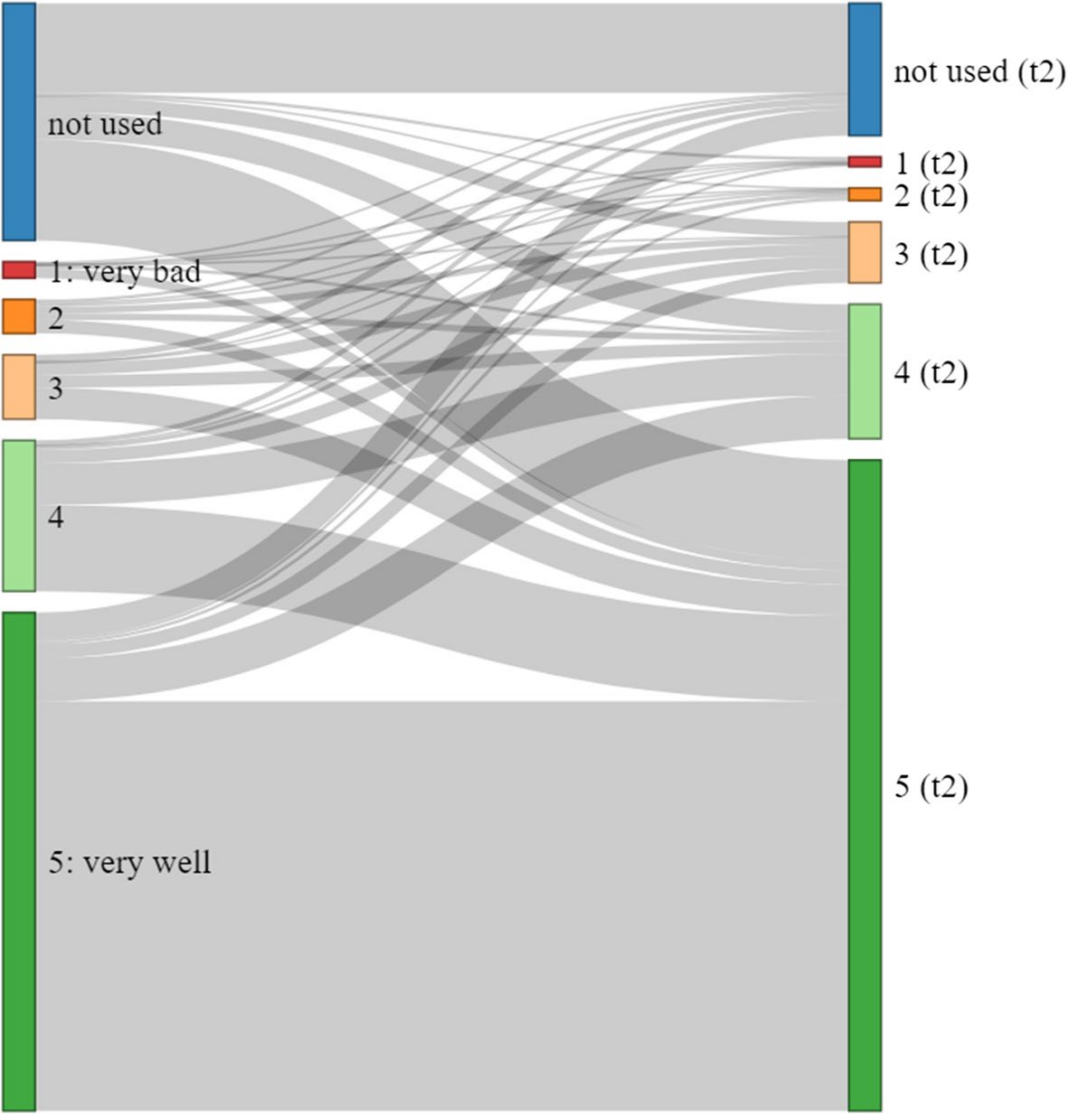
Extent of implementation of group separation indoors as reported by directors. Legend: changes in answers between first and second point of measurement, CKS-Survey 2020

But even when there are no significant changes in the mean values of a variable (e.g., similar assessment of the implementation across the ECEC centers), valuable information can still be obtained by analysing intra-individual changes within a person or institution (e.g., learning effects can be assumed in some cases). Figure 3 for instance shows how the frequency of value 4 (i.e., measures implemented to a “good” extent) does not change very much overall; however, only a small share of those reporting a 4 in the first survey also reported that answer in the second one. The prevalence of such intra-individual variance can be important for multivariate analyses, such as fixed-effects regression models which require a certain amount of variance on dependent or independent variables.

Furthermore, it can give hints as to how valid the used instrument is. In a situation as volatile as the ongoing pandemic, where pedagogical staff is constantly confronted with changing situations and challenges from parents and policymakers, differing answers at different points in time can be expected. Not finding such intra-individual changes could therefore be a sign of a lack of validity, since the instrument would then apparently measure some other aspect that is not influenced by the pandemic.

Other quality analyses are also possible by combining information from different sources: In the case of the CKS directors’ survey, parents and pedagogical staff of a subsample were also interviewed. This makes it possible to merge the respective data sets and compare the information given by different groups, thus gaining insights into the multi-perspective answering and into the reliability of the instruments.

For example, when comparing the directors survey (measurement point 2) and the staff survey (measurement point 1), which mostly took place within four weeks of one another, similarities as well as differences can be found: As to how well the observance of physical distancing between staff is working, there is no strong correlation between the information reported by staff and directors (Kendall’s tau = .03). Overall, directors see the implementation far more optimistic (mean 3.83 vs 2.57 on a scale from 1 to 5). However, smaller differences (3.87 vs. 3.48) and stronger correlations (Kendall’s tau = .20) can be observed when respondents are asked to rate how well physical distancing between staff and children of other groups is working. These results illustrate that pedagogical staff and directors can perceive situations quite differently, which has to be taken into account when analyses are conducted.

## Conclusion

We believe that our instruments are among the first to measure ECEC directors’ subjective level of information regarding pandemic-related regulations as well as hygiene and protective measures’ applicability, specifically in ECEC centers. These instruments can provide important information, for example concerning potential factors for the development of SARS-CoV-2 infections in ECEC settings (cf., Neuberger et al., 2021). The instruments were developed for the rather specific context of ECEC, yet they are broad enough to be combined with different dependent variables, such as diseases other than COVID-19. Therefore, we hope that our instruments will be useful for further research in the ECEC context, especially regarding health-related questions.

## Supplementary Information

**Additional file 1: Table 1.** ERiK/Corona-Kita-Survey constructs

**Additional file 2.** ERiK corona-specific add-on of directors’ questionnaire

**Additional file 3.** Corona-KiTa-Study directors’ questionnaire

**Additional file 4.** ERiK directors‘ questionnaire

**Additional file 5.** Question translations

**Additional file 6.** ERiK directors distribution compared to the KJH statistics

## Data Availability

The data of the ERiK study will be available in 2023, all questionnaires and further information are available online: www.dji.de/ERIK. Also, the Corona-KiTa-Study presents data and further information online: https://corona-kita-studie.de/. The discussed directors’ questionnaires of both studies are attached as additional material to the manuscript.

## Footnotes

1 The law “Gesetz zur Weiterentwicklung der Qualität und zur Verbesserung der Teilhabe in Tageseinrichtungen und in der Kindertagespflege (KiTa-Qualitäts- und -Teilhabeverbesserungsgesetz - KiQuTG)” describes dimensions of structural and in parts orientation quality along ten “fields of action” (in German: *Handlungsfelder*) plus measures on discharging parents from fees.

2 An indicator is a quantifiable characteristic of a defined population which has a standard definition (cf. Benova et al., 2020).

3 The ERiK-Surveys 2020 (Gedon et al., 2021) were conducted in 2020, and the ERiK-Surveys 2022 are in preparation and will be conducted in 2022. Data from the ERiK-Surveys 2020 and 2022 will be made publicly available at the DJI-FDZ (URL: 10.17621/erik2020) in early 2023.

4 The ERiK and CKS parent surveys are linked to the annual DJI Childcare Survey (KiBS) (Kuger/Gedon 2021) and will not be described further in the following.

5 Those care arrangements were just accessible for so-called “professions of societal relevance” (*systemrelevant*), like nurses, doctors or police officers.

6 This response time refers to the Corona add-on and the ERiK center director questionnaire developed for monitoring the KiQuTG (see Additional file 4).

7 However, no weighting procedure can eliminate selectivity, and it is clear that selectivity and non-response bias with respect to other unobserved characteristics could affect empirical data collected with the ERiK instrument. The comparison between the two datasets with regard to the distributions of the two socio-demographic variables is included in the Appendix, Additional file 6: ERiK directors distribution compared to the KJH statistics.

8 In addition, post-hoc power analyses (e.g., Häder & Häder, 2014) were conducted for the ERiK-Surveys 2020 to address the question of whether the sample size per state is large enough to provide accurate information, assuming a sampling error of 3 percent. For the directors survey, the achieved sample size was considered too small in Hamburg for meaningful analyses, with 57 completed questionnaires in relation to a population size of 1.163 directors in Hamburg according to the National Child and Youth Welfare Statistics (for more information, see Schacht et al. in prep). For the other federal states, the data on directors from the ERiK-Surveys 2020 even allows for a regional comparison—in addition to the multi-perspective comparison already presented. In next steps, ERiK plans on constructing relevant indicators for the field, analysing the non-response behaviour in more detail (based on the data from a non-response survey with providers and directors) and measuring the influence of the survey mode on the respondent responses (based on a random distribution of an online version versus a paper and online version of the instruments).
