# Supplementary material for: Establishing multi-perspective instruments in early education during COVID-19: measuring the implementation of protective measures and the subjective level of information about pandemic-related regulations
Source: Meas Instrum Soc Sci. 2022 May 12;4(1):7. doi: 10.1186/s42409-022-00033-2 (PMC9096761; doi:10.1186/s42409-022-00033-2)
Supplement: Supplementary file 2 — Additional file 2. ERiK corona-specific add-on of directors’ questionnaire [file 42409_2022_33_MOESM2_ESM.pdf]

# Zusatzfragen zur Corona-Lage

# **für die Einrichtungsleitung**

**Sehr geehrte Einrichtungsleitung,**

die Corona-Pandemie macht den hohen Stellenwert der Kindertagesbetreuung in Deutschland deutlich.

Sicher stehen auch Sie in Ihrer Einrichtung vor ungewöhnlichen Herausforderungen. Um die besonderen Bedingungen der aktuellen Situation abbilden zu können, haben wir entschieden, unsere für dieses Frühjahr geplante Befragung zu starten und dabei nicht nur nach der regulären Situation, sondern auch nach diesen besonderen Bedingungen zu fragen. Wir bitten Sie herzlich um Ihre Mitarbeit, damit wir die Rahmenbedingungen der Kindertagesbetreuung aus Sicht der Leitung gut beschreiben können.

**Am einfachsten können Sie die Fragen zur aktuellen Situation (zusammen mit dem Hauptfragebogen zur Studie) online ausfüllen.**

Bitte geben Sie hierzu folgende Adresse in Ihrem Internetbrowser ein:

| |
|---|
| |

Ihr persönlicher Zugangscode lautet:

| |
|---|
| |

Alternativ können Sie dieses Blatt ausfüllen und zusammen mit dem Hauptfragebogen zur Studie in dem beigefügten portofreien Rückumschlag an infas zurücksenden.

Deutsches Jugendinstitut

infas

infas Institut für angewandte Sozialwissenschaft GmbH

Postfach 240101
53154 Bonn
Tel. 0800/73 84 500
erik@infas.de

**1** **Wurde in Ihrer Einrichtung während der Maßnahmen zur Eindämmung des Corona-Virus ab Mitte März 2020 eine Notfallbetreuung für Kinder eingerichtet?**

| | | | |
|---|---|---|---|
| Ja, für Kinder von Eltern in systemrelevanten Berufsfeldern (z.B. Pflegekräfte, Polizisten) | ☐ | → | *Bitte weiter mit Frage 2* |
| Ja, für Kinder von Eltern in systemrelevanten Berufsfeldern und für Familien mit besonderen Belastungen | ☐ | → | *Bitte weiter mit Frage 2* |
| Nein | ☐ | → | *Bitte weiter mit Frage 3* |

**2** **Wie viele Kinder unter 6 Jahren haben durchschnittlich pro Tag das Angebot bei Ihnen in Anspruch genommen?**

☐ Kinder, die auch sonst regulär in der Einrichtung betreut werden

☐ Kinder, die sonst **nicht** regulär in der Einrichtung betreut werden

**3** **Wurde in diesem Zeitraum eine trägerübergreifende Notfallbetreuung in Ihrem Jugendamtsbezirk organisiert?**

| | |
|---|---|
| Ja | ☐ |
| Nein | ☐ |
| Weiß nicht | ☐ |

**4** **Wie gut fühlen Sie sich in der aktuellen Krise der Corona-Pandemie hinsichtlich folgender Aspekte informiert, um ein Betreuungsangebot in Ihrer Einrichtung bereithalten zu können?**

☞ *Bitte machen Sie in jeder Zeile eine Angabe, unabhängig davon ob Sie eine Notfallbetreuung bereithalten.*

| | Sehr schlecht 1 | 2 | 3 | 4 | 5 | Sehr gut 6 |
|---|---|---|---|---|---|---|
| Bezogen auf den Schutz des Personals | ☐ | ☐ | ☐ | ☐ | ☐ | ☐ |
| Bezogen auf Schutzvorkehrungen für die Kinder | ☐ | ☐ | ☐ | ☐ | ☐ | ☐ |
| Bezogen auf Informationen für die Eltern | ☐ | ☐ | ☐ | ☐ | ☐ | ☐ |
| Bezogen auf gesundheitliche Vorsorge (z.B. Aushändigen von Plakaten o.ä. mit den Grundregeln der Hygiene, Aushändigen von Desinfektionsmitteln, Handschuhen) | ☐ | ☐ | ☐ | ☐ | ☐ | ☐ |
| Bezogen auf Informationen/Regelungen dazu, ab wann eine Fachkraft nicht mehr eingesetzt darf | ☐ | ☐ | ☐ | ☐ | ☐ | ☐ |
| Bezogen auf Ausfallzahlungen, wenn Kinder nicht betreut werden können | ☐ | ☐ | ☐ | ☐ | ☐ | ☐ |

**5** **Wie gut funktioniert aus Ihrer Sicht in dieser herausfordernden Zeit die Zusammenarbeit der verschiedenen Akteure (u.a. Jugendamt, Landesbehörden, Träger)?**

☞ *Bitte machen Sie in jeder Zeile eine Angabe.*

| | Sehr schlecht 1 | 2 | 3 | 4 | 5 | Sehr gut 6 |
|---|---|---|---|---|---|---|
| Bei der Organisation eines Betreuungsangebotes? | ☐ | ☐ | ☐ | ☐ | ☐ | ☐ |
| Bei der Regelung der Kosten für die Eltern? | ☐ | ☐ | ☐ | ☐ | ☐ | ☐ |

Im beiliegenden Fragebogen zur Studie bitten wir Sie abweichend von diesen Fragen nun, sich bei allen Antworten auf die „normale“ bzw. typische Situation **bis Mitte März 2020** zu beziehen. Das bedeutet, bitte beantworten Sie die Fragen in Bezug auf die Situation bevor Ihr Alltag durch das Corona-Virus beeinflusst wurde.



7135/2020
