# Supplementary material for: Establishing multi-perspective instruments in early education during COVID-19: measuring the implementation of protective measures and the subjective level of information about pandemic-related regulations
Source: Meas Instrum Soc Sci. 2022 May 12;4(1):7. doi: 10.1186/s42409-022-00033-2 (PMC9096761; doi:10.1186/s42409-022-00033-2)
Supplement: Supplementary file 3 — Additional file 3. Corona-KiTa-Study directors’ questionnaire [file 42409_2022_33_MOESM3_ESM.pdf]

Corona-KiTa-Studie

ERiK

Fragebogen
für die Einrichtungsleitung

Sehr geehrte Einrichtungsleitung,

die Corona-Pandemie hat die Bedeutung der Kindertagesbetreuung in den letzten Monaten besonders hervorgehoben. Gleichzeitig stellt die Situation sicherlich auch Sie und Ihre Mitarbeiterinnen und Mitarbeiter vor zahlreiche Herausforderungen.

In der deutschlandweiten Corona-KiTa-Studie, die in Kooperation mit dem ERiK-Projekt durchgeführt wird, interessieren wir uns im Besonderen für Ihre Perspektive als Einrichtungsleitung hinsichtlich der Fragen, vor welche Schwierigkeiten die Pandemie Sie stellt und welche Lösungsansätze Sie entwickeln.

Ihre Unterstützung ist für den Erfolg der Studie dabei von größter Bedeutung. Ihre Angaben werden nur in anonymisierter Form, das heißt ohne Namen und Adresse, und nur zusammengefasst mit den Angaben der anderen Befragten ausgewertet. Alle Regeln des Datenschutzes werden vollständig eingehalten.

Am einfachsten können Sie den Fragebogen online ausfüllen.

Bitte geben Sie hierzu folgende Adresse in Ihrem Internetbrowser ein:

Ihr persönlicher Zugangscode lautet:

Alternativ zu dem Online-Fragebogen können Sie diesen schriftlichen Fragebogen ausfüllen und in dem beigefügten portofreien Rückumschlag an infas zurücksenden.

Wie ist der Fragebogen auszufüllen?

Kreuzen Sie bitte die jeweils zutreffenden Antwortmöglichkeiten in den dafür vorgesehenen Kästchen an: ☒

In die großen Kästchen setzen Sie bitte die jeweils erfragten Zahlen oder Angaben ein: 87

Bitte achten Sie auf entsprechende Hinweise zum Ausfüllen von Fragen: ☞ *Bitte machen Sie nur eine Angabe.*

Bitte achten Sie auf entsprechende Hinweise zum Überspringen von Fragen: → ***Bitte weiter mit Frage xy***

DJI Deutsches Jugendinstitut

infas

infas Institut für angewandte Sozialwissenschaft GmbH

Postfach 240101
53154 Bonn
Tel. 0800/73 84 500
corona-kita@infas.de

7370/2020/L

1 In welchem Umfang bietet Ihre Kindertageseinrichtung derzeit Betreuung an?

☞ *Bitte geben Sie alles Zutreffende an.*

- Wir bieten derzeit Regelbetrieb unter Pandemiebedingungen an. ☐
- Wir bieten pandemiebedingt nur einen eingeschränkten Betreuungsbetrieb an (z.B. weniger Kinder oder Betreuungsstunden als im Regelbetrieb unter Pandemiebedingungen) ☐
- Eine Gruppe/mehrere Gruppen ist/sind derzeit aufgrund von Verdachts- oder Infektionsfällen geschlossen. ☐
- Die gesamte Einrichtung ist derzeit aufgrund von Verdachts- oder Infektionsfällen geschlossen. ☐

2 Wie viele Verdachts- oder bestätigte Infektionsfälle einer SARS-CoV-2-Infektion (COVID-19) haben Sie derzeit jeweils in Ihrer Einrichtung?

☞ *Wenn es derzeit keinen entsprechenden Fall in Ihrer Einrichtung gibt, tragen Sie bitte eine 0 ein.*

	Anzahl Verdachtsfälle	Anzahl bestätigte Infektionsfälle
bei Kindern	☐	☐
bei MitarbeiterInnen oder Eltern	☐	☐

3 Mit welchem Gruppenkonzept arbeiten Sie derzeit in Ihrer Kindertageseinrichtung?

- Offen 1 ☐
- Teilweise offen 2 ☐
- Feste Gruppenstruktur 3 ☐

4 Wie viele Stunden pro Woche hat Ihre Kindertageseinrichtung derzeit geöffnet?

☐ Stunden pro Woche

5 Wie viele Kinder werden derzeit in Ihrer Kindertageseinrichtung betreut?

☞ *Bitte geben Sie die Anzahl der Kinder an, die derzeit in der Regel anwesend sind.*
Wenn Sie keine Kinder in einer der Altersgruppen haben, geben Sie bitte jeweils „0“ ein.

☐ Anzahl Kinder unter 3 Jahren

☐ Anzahl Kinder ab 3 Jahren bis zum Schuleintritt

☐ Anzahl Grundschulkinder

6 Wie viele Gruppen gibt es derzeit in Ihrer Kindertageseinrichtung?

☞ *Wenn Sie keine Gruppen in einer der Altersspannen haben, geben Sie bitte jeweils „0“ ein.*

☐ Anzahl Krippengruppen (0 bis unter 3 Jahre)

☐ Anzahl Kindergartengruppen (3 bis 7 Jahre)

☐ Anzahl Gruppen Schulkinder

☐ Anzahl altersgemischte Gruppen ohne Schulkinder (0 bis 7 Jahre)

☐ Anzahl altersgemischte Gruppen mit Schulkindern (0 bis 12 Jahre)

7370/2020

7 Wie viele Sanitärbereiche für Kinder gibt es in Ihrer Kindertageseinrichtung?

☞ *Als Sanitärbereich gilt ein abgetrennter Bereich mit (ggf. mehreren) Waschbecken/Toiletten/ Wickelbereich.*

☐ Anzahl Sanitärbereiche

8 Sind in Ihrer Kindertageseinrichtung mehr als 6 pädagogische MitarbeiterInnen (inkl. Leitungen) beschäftigt?

Ja	1 ☐	→ ***Bitte weiter mit Frage 9***
Nein	0 ☐	→ ***Bitte weiter mit Frage 12***

9 Gibt es in Ihrer Kindertageseinrichtung derzeit Personalengpässe beim pädagogischen Personal?

☞ *Bitte geben Sie alles Zutreffende an.*

Ja, aufgrund der Zugehörigkeit der Beschäftigten zu Corona-Risikogruppen	☐
Ja, aufgrund der Zugehörigkeit von Haushaltsmitgliedern der Beschäftigten zu Corona-Risikogruppen	☐
Ja, aufgrund von Verdachtsfällen oder COVID-19-bedingter Quarantäne bei Beschäftigten oder deren Haushaltsmitgliedern	☐
Ja, aufgrund von Krankheit (außer Corona)/Beschäftigungsverbot bei Schwangerschaft/ Mutterschutz	☐
Ja, aufgrund von pandemiebedingten Betreuungsproblemen bei den Beschäftigten mit Kindern	☐
Ja, aufgrund von Abwesenheit der Beschäftigten durch Urlaub	☐
Ja, aufgrund von Abwesenheit der Beschäftigten durch Fort- und Weiterbildungen	☐
Ja, aufgrund von hohem Teilzeitaufkommen unter den Beschäftigten	☐
Ja, aufgrund von unbesetzten Stellen	☐
Ja, aus sonstigen Gründen	☐
Wir haben derzeit keine Personalengpässe in unserer Einrichtung.	☐

10 Haben Sie pädagogische MitarbeiterInnen, die zu einer Corona-Risikogruppe (z.B. älter als 60 Jahre oder mit Vorerkrankungen wie Herzkreislauferkrankung, Diabetes, etc. oder RaucherInnen) gehören?

Ja	1 ☐	→ ***Bitte weiter mit Frage 11***
Nein	0 ☐	→ ***Bitte weiter mit Frage 12***

11 Für wie viele Ihrer pädagogischen MitarbeiterInnen, die zu einer Corona-Risikogruppe gehören, treffen jeweils folgende Situationen zu?

☞ *Bitte machen Sie in jeder Zeile eine Angabe.*

	(Fast) alle 1	Einige 2	Keine 3
Sie arbeiten im Gruppendienst mit Kindern.	☐	☐	☐
Sie arbeiten derzeit im Homeoffice.	☐	☐	☐
Sie sind derzeit krank geschrieben.	☐	☐	☐

Einige der folgenden Fragen beziehen sich auf die Zeit des „Lockdowns“. Damit ist die Zeit nach der 12. Kalenderwoche mit der Schließung der Kindertageseinrichtungen (16.03.2020) bis zu deren sukzessiven Öffnung im Sommer 2020 gemeint.

12 **Wie häufig wurden in Ihrer Kindertageseinrichtung während des Lockdowns im Frühjahr 2020 folgende Kommunikationswege beim Kontakt zu Eltern genutzt?**

☞ *Bitte machen Sie in jeder Zeile eine Angabe.*

	Nie (0)	Seltener als 1-2 Mal im Monat (1)	1-2 Mal im Monat (2)	1-2 Mal pro Woche (3)	3-4 Mal pro Woche (4)	(Fast) täglich (5)
E-Mail	☐	☐	☐	☐	☐	☐
Briefe	☐	☐	☐	☐	☐	☐
Telefon	☐	☐	☐	☐	☐	☐
Textnachrichten (z.B. SMS, WhatsApp, etc.)	☐	☐	☐	☐	☐	☐
Videochat (z.B. Skype, Zoom, etc.)	☐	☐	☐	☐	☐	☐
Soziale Netzwerke (z.B. Facebook, etc.)	☐	☐	☐	☐	☐	☐
Selbstgedrehte Videos (z.B. Aufführung eines Puppentheaters)	☐	☐	☐	☐	☐	☐
Aufgenommene Videobotschaften (z.B. Grüße)	☐	☐	☐	☐	☐	☐
Persönlicher Kontakt unter Beachtung von ausreichendem Abstand	☐	☐	☐	☐	☐	☐
Persönlicher Kontakt ohne ausreichenden Abstand	☐	☐	☐	☐	☐	☐

7370/2020

13 Wie häufig werden in Ihrer Kindertageseinrichtung <u>derzeit</u> folgende Kommunikationswege beim Kontakt zu Eltern genutzt?

☞ Bitte machen Sie in jeder Zeile eine Angabe.

	Nie<br>0	Seltener als 1-2 Mal im Monat<br>1	1-2 Mal im Monat<br>2	1-2 Mal pro Woche<br>3	3-4 Mal pro Woche<br>4	(Fast) täglich<br>5
E-Mail	☐	☐	☐	☐	☐	☐
Briefe	☐	☐	☐	☐	☐	☐
Telefon	☐	☐	☐	☐	☐	☐
Textnachrichten (z.B. SMS, WhatsApp, etc.)	☐	☐	☐	☐	☐	☐
Videochat (z.B. Skype, Zoom, etc.)	☐	☐	☐	☐	☐	☐
Soziale Netzwerke (z.B. Facebook, etc.)	☐	☐	☐	☐	☐	☐
Selbstgedrehte Videos (z.B. Aufführung eines Puppentheaters)	☐	☐	☐	☐	☐	☐
Aufgenommene Videobotschaften (z.B. Grüße)	☐	☐	☐	☐	☐	☐
Persönlicher Kontakt unter Beachtung von ausreichendem Abstand	☐	☐	☐	☐	☐	☐
Persönlicher Kontakt ohne ausreichenden Abstand	☐	☐	☐	☐	☐	☐

14 Inwieweit ist Ihre Kindertageseinrichtung mit folgenden Medien ausgestattet?

☞ Bitte machen Sie in jeder Zeile eine Angabe. Nicht gemeint sind Privatgeräte der Beschäftigten.

	Vorhanden und es besteht kein weiterer Bedarf<br>3	Vorhanden aber es besteht weiterer Bedarf<br>2	Nicht vorhanden aber es besteht Bedarf<br>1	Nicht vorhanden und es besteht kein Bedarf<br>0
Festnetztelefon	☐	☐	☐	☐
Internetzugang	☐	☐	☐	☐
Computer/Laptop	☐	☐	☐	☐
Smartphone	☐	☐	☐	☐
Tablet	☐	☐	☐	☐
Digitalkamera	☐	☐	☐	☐
Videokamera	☐	☐	☐	☐

15 **Welche organisatorischen Maßnahmen wurden/werden in Ihrer Kindertageseinrichtung angewandt/angewendet?**

☞ Bitte geben Sie in jeder Zeile alles Zutreffende an.

	Vor dem Lockdown 1	Während des Lockdowns 2	Derzeit 3	Noch nie 4
Verkürzte Öffnungszeiten	☐	☐	☐	☐
Platz-Sharing unter Kindern (z.B. vor- und nachmittags, tageweise, wochenweise)	☐	☐	☐	☐
Reduktion individueller Betreuungszeiten	☐	☐	☐	☐
Neue Gruppenbildung aufgrund von eingeschränktem Betreuungsbetrieb	☐	☐	☐	☐
Aufnahme von Gästekindern (z.B. vom Personal)	☐	☐	☐	☐
Einsatz von Springerkräften aus der eigenen Einrichtung	☐	☐	☐	☐
Einsatz von Springerkräften aus anderen Einrichtungen	☐	☐	☐	☐

16 **Werden bei Ihnen derzeit folgende Aktionen durchgeführt und wenn ja, in welcher Form?**

☞ Bitte geben Sie in jeder Spalte alles Zutreffende an.

	Persönlich	Telefonisch	Virtuell
Entwicklungsgespräche	☐	☐	☐
Elternabende	☐	☐	☐
Infoabende für Eltern (z.B. mit externen ReferentInnen)	☐	☐	☐
Teambesprechungen	☐	☐	☐
Teilnahme an Supervisionen	☐	☐	☐
Teilnahme an Fortbildungen	☐	☐	☐

17 **Wie häufig werden bei Ihnen derzeit folgende Gespräche durchgeführt?**

☞ Bitte machen Sie in jeder Zeile eine Angabe.

	Nie 0	1	2	3	4	Sehr häufig 5
Kurze Gespräche mit Eltern innerhalb der Räumlichkeiten (z.B. Tür- und Angelgespräche)	☐	☐	☐	☐	☐	☐
Kurze Gespräche mit Eltern außerhalb der Räumlichkeiten (z.B. Tür- und Angelgespräche)	☐	☐	☐	☐	☐	☐
Längere Gespräche mit Eltern innerhalb der Räumlichkeiten (z.B. Entwicklungsgespräch)	☐	☐	☐	☐	☐	☐
Längere Gespräche mit Eltern außerhalb der Räumlichkeiten (z.B. Entwicklungsgespräch)	☐	☐	☐	☐	☐	☐

18 Setzen Sie derzeit folgende Maßnahmen um?

☞ Bitte geben Sie in jeder Zeile alles Zutreffende an.

	Umsetzung? Ja [1]	Umsetzung? Nein [2]	Warum wird diese Maßnahme derzeit nicht umgesetzt? Kein Bedarf	Keine finanziellen Mittel	Keine personellen Ressourcen
Fort-/Weiterbildungen zum Umgang mit digitalen Medien	☐	☐ →	☐	☐	☐
Fort-/Weiterbildungen zum Thema Hygienemaßnahmen in Kitas	☐	☐ →	☐	☐	☐
Fort-/Weiterbildungen zum Thema Datenschutz	☐	☐ →	☐	☐	☐
Anschaffung von Tablet-PCs, Computer, etc.	☐	☐ →	☐	☐	☐
Umgestaltung des Innenbereichs	☐	☐ →	☐	☐	☐
Umgestaltung des Außenbereiches	☐	☐ →	☐	☐	☐
Anschaffung zusätzlicher pädagogischer Materialien (z.B. Spielzeug, Bücher)	☐	☐ →	☐	☐	☐
Anschaffung zusätzlicher Möbel	☐	☐ →	☐	☐	☐

19 Wie gut funktioniert die Umsetzung der folgenden Maßnahmen in Ihrer Einrichtung ?

☞ Bitte machen Sie in jeder Zeile eine Angabe. Falls Sie eine Maßnahme in Ihrer Einrichtung derzeit nicht anwenden, geben Sie bitte „trifft nicht zu" an.

	Sehr schlecht 1	2	3	4	Sehr gut 5	Trifft nicht zu [9]
Regelmäßiges Durchlüften	☐	☐	☐	☐	☐	☐
Regelmäßige Desinfektion von Möbeln, Türgriffen oder Spielzeug	☐	☐	☐	☐	☐	☐
Tägliche Temperaturmessung bei Kindern	☐	☐	☐	☐	☐	☐
Tägliche Temperaturmessung beim Personal	☐	☐	☐	☐	☐	☐
Regelmäßiges Händewaschen mit den Kindern	☐	☐	☐	☐	☐	☐
Regelmäßiges Händewaschen beim Personal	☐	☐	☐	☐	☐	☐
Regelmäßiges Testen der Beschäftigten auf COVID-19	☐	☐	☐	☐	☐	☐
Eltern tragen Mund- und Nasenschutz (auch Face Shields) beim Kontakt mit der Einrichtung.	☐	☐	☐	☐	☐	☐
Das pädagogische Personal trägt in bestimmten Situationen Mund- und Nasenschutz.	☐	☐	☐	☐	☐	☐
Laufwege in der Einrichtung wurden markiert und werden genutzt (z.B. für Eltern oder Kinder unterschiedliche Gruppen).	☐	☐	☐	☐	☐	☐
Beschäftigte halten Distanz zueinander.	☐	☐	☐	☐	☐	☐
Feste Zuweisung Personal zu Gruppe	☐	☐	☐	☐	☐	☐
Gruppentrennung im Innenbereich	☐	☐	☐	☐	☐	☐
Gruppentrennung im Außenbereich	☐	☐	☐	☐	☐	☐
Beschäftigte halten Distanz zu Kindern ihrer Gruppe.	☐	☐	☐	☐	☐	☐
Beschäftigte halten Distanz zu Kindern anderer Gruppen.	☐	☐	☐	☐	☐	☐

20 Inwieweit tragen pädagogische MitarbeiterInnen Mund- und Nasenschutz?

☞ Bitte machen Sie in jeder Zeile eine Angabe.

**Die pädagogischen MitarbeiterInnen tragen einen Mund- und Nasenschutz …**	**Nie 0**	**1**	**2**	**3**	**4**	**Immer 5**
beim Kontakt mit Eltern.	☐	☐	☐	☐	☐	☐
beim Kontakt mit KollegInnen (z.B. in Pausenräumen, Besprechungen).	☐	☐	☐	☐	☐	☐
beim Kontakt mit sonstigen Externen (z.B. Lieferanten, Therapeuten...).	☐	☐	☐	☐	☐	☐
beim Wickeln/Begleiten des Toilettengangs.	☐	☐	☐	☐	☐	☐
bei der pädagogischen Arbeit in der Gruppe.	☐	☐	☐	☐	☐	☐
im Kontakt mit Kindern, wenn keine Distanz gehalten werden kann (z.B. beim gemeinsamen Buch anschauen).	☐	☐	☐	☐	☐	☐
in sonstigen Situationen.	☐	☐	☐	☐	☐	☐

21 Inwieweit wurden/werden folgende Maßnahmen zur Organisation der gemeinsamen Nutzung des Sanitär- oder Wickelbereichs vor und während des Lockdowns sowie derzeit angewendet?

☞ Bitte geben Sie in jeder Spalte alles Zutreffende an.

	**Vor dem Lockdown** 1	**Während des Lockdowns** 2	**Derzeit** 3	**Noch nie** 4
Alle Kinder wurden/werden beim Toilettengang begleitet.	☐	☐	☐	☐
In den Sanitärbereichen wurden/werden Toiletten, Waschbecken oder Wickelbereiche einzelnen Gruppen zugewiesen.	☐	☐	☐	☐
Die Anzahl der erlaubten Personen im Sanitärbereich wurde/ist festgelegt.	☐	☐	☐	☐
Kinder mussten/müssen im Sanitärbereich Abstand halten.	☐	☐	☐	☐
Der Sanitärbereich wurde/wird mindestens zwei Mal täglich gereinigt.	☐	☐	☐	☐

7370/2020

22 Wie wurden Essenssituationen in Ihrer Einrichtung vor und während des Lockdowns gestaltet? Wie sieht es derzeit aus?

Bitte geben Sie in jeder Spalte alles Zutreffende an.

	Vor dem Lockdown 1	Während des Lockdowns 2	Derzeit 3	Noch nie 4
Das Mittagessen fand/findet im Gruppenraum getrennt nach Gruppen statt.	☐	☐	☐	☐
Das Mittagessen fand/findet in extra dafür vorgesehen Räumen statt (z.B. Bistro, Kinderrestaurant).	☐	☐	☐	☐
Beim Mittagessen wurde/wird ein Mindestabstand eingehalten (z.B. durch eine entsprechende Stuhlreihung).	☐	☐	☐	☐
Kinder durften/dürfen sich das Essen selbst nehmen.	☐	☐	☐	☐
Das Essen wurde/wird kontaktlos an die Gruppe übergeben (z.B. auf einem Servierwagen vor die Tür gestellt).	☐	☐	☐	☐
Kinder durften/dürfen ihr Essen teilen.	☐	☐	☐	☐
Kinder durften/dürfen ihre Becher/Trinkflaschen teilen.	☐	☐	☐	☐
Kinder durften/dürfen bei der Zubereitung der Brotzeit oder des Mittagessens mithelfen.	☐	☐	☐	☐

23 Haben Sie einen Außenbereich?

Ja 1 ☐ → *Bitte weiter mit Frage 24*

Nein 0 ☐ → *Bitte weiter mit Frage 25*

24 Wie ist derzeit die Nutzung Ihres Außenbereiches geregelt?

Bitte geben Sie alles Zutreffende an.

- Jede Gruppe hat ihren festen Bereich auf dem Außengelände. ☐
- Die Gruppen nutzen denselben Bereich gleichzeitig. ☐
- Die Gruppen nutzen denselben Bereich im Wechsel (z.B. nach vereinbarten Zeiten). ☐
- Auf dem Außengelände gelten Abstandsregeln zwischen Kindern verschiedener Gruppen. ☐
- Der Außenbereich wurde aufgrund der Corona-Pandemie aufgeteilt und getrennt (z.B. durch Flatterbänder/Absperrungen). ☐

25 Wo findet derzeit die Übergabe der Kinder bei der Bring- und Abholsituation statt?

Bitte geben Sie alles Zutreffende an.

- Außerhalb des Kita-Gebäudes (z.B. vor der Tür, im Garten, an der Straße) ☐
- Im Eingangsbereich der Kita (z.B. im Flur, im Windfang) ☐
- In der Kita, aber nicht im Gruppenraum, z.B. in der Garderobe oder vor der Gruppentür ☐
- In der Kita, im Gruppenraum ☐

26 **Welche der folgenden Aspekte treffen in Bezug auf die Eingewöhnung derzeit auf Ihre Einrichtung zu?**

☞ *Bitte geben Sie alles Zutreffende an.*

Die Eingewöhnung läuft so ab wie vor Corona.	☐
Eltern dürfen mit Mund-Nasenschutz im Gruppenraum anwesend sein.	☐
Es wird darauf geachtet, dass Eltern möglichst wenig in der Einrichtung anwesend sind.	☐
Im Herbst 2020 wurden Eingewöhnungen aus dem Frühjahr nachgeholt, die pandemiebedingt ausgesetzt werden mussten.	☐
Eingewöhnungen sind derzeit pandemiebedingt ausgesetzt.	☐

27 **Bereiten Ihnen derzeit folgende Aufgaben Schwierigkeiten?**

☞ *Bitte machen Sie in jeder Zeile eine Angabe.*
Falls Sie eine Maßnahme in Ihrer Einrichtung derzeit nicht anwenden, geben Sie bitte „trifft nicht zu" an.

	Keine Schwierig-keiten 0	1	2	3	Sehr große Schwierig-keiten 4	Trifft nicht zu 9
Informationsbeschaffung über geltende Schutz- und Hygienemaßnahmen	☐	☐	☐	☐	☐	☐
Umsetzung geltender Schutz- und Hygienemaßnahmen	☐	☐	☐	☐	☐	☐
Beschaffung von Hygieneartikeln, wie Desinfekions-spendern/Desinfektionsmitteln	☐	☐	☐	☐	☐	☐
Beschaffung technischer Geräte, wie z.B. Tablets, für den Austausch mit Kindern oder Eltern	☐	☐	☐	☐	☐	☐
Korrekte Anwendung von Datenschutzrichtlinien (z.B. bezüglich der Weitergabe von Kindervideos)	☐	☐	☐	☐	☐	☐
Auswahl der Kinder, die Anspruch auf eingeschränkte Betreuung haben (z.B. bzgl. systemrelevanter Berufe)	☐	☐	☐	☐	☐	☐
Betreuung von Kindern mit Schnupfen	☐	☐	☐	☐	☐	☐
Organisation des eingeschränkten Betriebs	☐	☐	☐	☐	☐	☐
Personalauswahl und -planung für den eingeschränkten Betrieb	☐	☐	☐	☐	☐	☐
Kontakt zu den Eltern (Information der Eltern, Anfragen von Eltern)	☐	☐	☐	☐	☐	☐
Raumplanung und Umgestaltung	☐	☐	☐	☐	☐	☐
Schaffung von Akzeptanz bei den Eltern hinsichtlich neuer Regelungen	☐	☐	☐	☐	☐	☐
Organisation der Reinigung von Räumlichkeiten	☐	☐	☐	☐	☐	☐
Der Betreuungsnachfrage gerecht werden	☐	☐	☐	☐	☐	☐

7370/2020

28 **Welche Regeln gelten <u>derzeit</u> in Ihrer Einrichtung für <u>Kinder</u> mit leichtem Schnupfen oder Husten?**

☞ *Bitte machen Sie in jeder Spalte eine Angabe.*

	Kinder mit leichtem... Schnupfen 1	Husten 2
dürfen betreut werden, wenn nach 24-48 Stunden keine weiteren Symptome hinzukommen.	☐	☐
dürfen nicht betreut werden, bis die Symptome abgeklungen sind.	☐	☐
dürfen nach ärztlicher Untersuchung durch Kinderarzt betreut werden (ohne Attest).	☐	☐
dürfen nach ärztlicher Untersuchung durch Kinderarzt und Attest („Gesundschreibung") betreut werden.	☐	☐

29 **Welche Regeln gelten <u>derzeit</u> in Ihrer Einrichtung für <u>pädagogische MitarbeiterInnen</u> mit leichtem Schnupfen oder Husten?**

☞ *Bitte machen Sie in jeder Spalte eine Angabe.*

	Pädagogische MitarbeiterInnen mit leichtem... Schnupfen 1	Husten 2
dürfen zur Arbeit kommen, wenn nach 24-48 Stunden keine weiteren Symptome hinzukommen.	☐	☐
dürfen nicht zur Arbeit kommen, bis die Symptome abgeklungen sind.	☐	☐
dürfen nach ärztlicher Untersuchung/Rücksprache mit dem Arzt arbeiten (ohne Attest).	☐	☐
dürfen nach ärztlicher Untersuchung/Rücksprache mit dem Arzt und Attest („Gesundschreibung") arbeiten.	☐	☐
dürfen nur nach Durchführung eines Tests auf COVID-19 arbeiten (negatives Testergebnis).	☐	☐

30 **Wie bewerten Sie die Ausstattung folgender Räume hinsichtlich der Erfüllung notwendiger Schutz- und Hygienemaßnahmen?**

☞ *Bitte machen Sie in jeder Zeile eine Angabe.*
Falls Ihre Einrichtung über eine bestimmte Raumart nicht verfügt, geben Sie bitte „gibt es nicht" an.

	Sehr schlecht 1	2	3	4	Sehr gut 5	Gibt es nicht 9
Gruppenräume	☐	☐	☐	☐	☐	☐
Turnräume/Bewegungsräume	☐	☐	☐	☐	☐	☐
Räume zum Essen (z.B. Bistro, Kinderrestaurant)	☐	☐	☐	☐	☐	☐
Sanitärbereiche (Toiletten/Wickelbereiche)	☐	☐	☐	☐	☐	☐
Schlafräume	☐	☐	☐	☐	☐	☐
Andere Funktionsräume (z.B. Atelier, Musikraum, Werkstatt)	☐	☐	☐	☐	☐	☐
Andere Räume, z.B. Nebenräume	☐	☐	☐	☐	☐	☐

31 Wie beurteilen Sie die Qualität der Interaktionen vor dem Lockdown, während des Lockdowns und derzeit?

☞ *Bitte machen Sie in jeder Zeile eine Angabe.*

Zusammenspiel Kind/Kind	Sehr schlecht 1	2	3	4	Sehr gut 5
Vor dem Lockdown	☐	☐	☐	☐	☐
Während des Lockdowns	☐	☐	☐	☐	☐
Derzeit	☐	☐	☐	☐	☐

Betreuungsqualität Fachkraft/Kind	Sehr schlecht 1	2	3	4	Sehr gut 5
Vor dem Lockdown	☐	☐	☐	☐	☐
Während des Lockdowns	☐	☐	☐	☐	☐
Derzeit	☐	☐	☐	☐	☐

Kooperation Eltern/Fachkraft	Sehr schlecht 1	2	3	4	Sehr gut 5
Vor dem Lockdown	☐	☐	☐	☐	☐
Während des Lockdowns	☐	☐	☐	☐	☐
Derzeit	☐	☐	☐	☐	☐

32 Inwieweit haben folgende Aktivitäten während des Lockdowns im Vergleich zum Regelbetrieb vor dem Lockdown im pädagogischen Alltag ab-/zugenommen?

☞ *Bitte machen Sie in jeder Zeile eine Angabe.*
Wenn Sie bestimmte Aktivitäten weder im Regelbetrieb noch im Lockdown angeboten haben, dann geben Sie bitte „gab es nicht“ an.

	Stark abgenommen 1	2	3	4	Stark zugenommen 5	Gab es nicht 9
Anpassung der Arbeit der pädagogischen Fachkräfte an die Bedürfnisse der Kinder	☐	☐	☐	☐	☐	☐
Individualisiertes Arbeiten (pädagogische Situationen mit einer Fachkraft und einem Kind)	☐	☐	☐	☐	☐	☐
Projektarbeit	☐	☐	☐	☐	☐	☐
Erledigen pflegerischer Aufgaben	☐	☐	☐	☐	☐	☐
Beobachtung und Dokumentation der kindlichen Entwicklung	☐	☐	☐	☐	☐	☐

33 Inwieweit bieten sie die folgenden Aktivitäten derzeit an?

☞ *Bitte machen Sie in jeder Zeile eine Angabe.*
Falls Ihre Einrichtung eine bestimmte Aktivität derzeit nicht anbietet, geben Sie bitte „gibt es nicht“ an.

	Sehr selten 1	2	3	4	Sehr häufig 5	Gibt es nicht 9
Anpassung der Arbeit der pädagogischen Fachkräfte an die Bedürfnisse der Kinder	☐	☐	☐	☐	☐	☐
Individualisiertes Arbeiten (pädagogische Situationen mit einer Fachkraft und einem Kind)	☐	☐	☐	☐	☐	☐
Projektarbeit	☐	☐	☐	☐	☐	☐
Erledigen pflegerischer Aufgaben	☐	☐	☐	☐	☐	☐
Beobachtung und Dokumentation der kindlichen Entwicklung	☐	☐	☐	☐	☐	☐

7370/2020

34 Inwieweit haben die folgenden pädagogischen Aufgaben <u>während des Lockdowns im Vergleich zum Regelbetrieb vor dem Lockdown</u> an Bedeutung im pädagogischen Alltag ab-/zugenommen?

☞ Bitte machen Sie in jeder Zeile eine Angabe.
Falls Sie bestimmte Aufgaben weder im Regelbetrieb noch im Lockdown nachkommen konnten, dann geben Sie bitte „gab es nicht“ an.

	Stark abgenommen 1	2	3	4	Stark zugenommen 5	Gab es nicht 9
Sprachliche Bildung	☐	☐	☐	☐	☐	☐
MINT (Mathematik, Informatik, Naturwissenschaften, Technik)	☐	☐	☐	☐	☐	☐
Förderung von Motorik/Bewegung	☐	☐	☐	☐	☐	☐
Musikalisch-künstlerische Bildung	☐	☐	☐	☐	☐	☐
Kompetenzförderung im Umgang mit digitalen Medien	☐	☐	☐	☐	☐	☐
Gesundheitsförderung (z.B. Förderung gesunder Ernährung)	☐	☐	☐	☐	☐	☐
Förderung der sozial-emotionalen Entwicklung	☐	☐	☐	☐	☐	☐
Inklusion	☐	☐	☐	☐	☐	☐
Interkulturelle Arbeit	☐	☐	☐	☐	☐	☐
Umsetzung von Kinderrechten (z.B. Mitbestimmung, Beschwerdemöglichkeiten)	☐	☐	☐	☐	☐	☐
Vorschule	☐	☐	☐	☐	☐	☐
Zusammenarbeit mit Familien/Erziehungspartnerschaft	☐	☐	☐	☐	☐	☐

35 Inwieweit können Sie den folgenden pädagogischen Aufgaben <u>derzeit</u> nachkommen?

☞ Bitte machen Sie in jeder Zeile eine Angabe.

	Gar nicht 0	Sehr selten 1	2	3	4	Sehr häufig 5
Sprachliche Bildung	☐	☐	☐	☐	☐	☐
MINT (Mathematik, Informatik, Naturwissenschaften, Technik)	☐	☐	☐	☐	☐	☐
Förderung von Motorik/Bewegung	☐	☐	☐	☐	☐	☐
Musikalisch-künstlerische Bildung	☐	☐	☐	☐	☐	☐
Kompetenzförderung im Umgang mit digitalen Medien	☐	☐	☐	☐	☐	☐
Gesundheitsförderung (z.B. Förderung gesunder Ernährung)	☐	☐	☐	☐	☐	☐
Förderung der sozial-emotionalen Entwicklung	☐	☐	☐	☐	☐	☐
Inklusion	☐	☐	☐	☐	☐	☐
Interkulturelle Arbeit	☐	☐	☐	☐	☐	☐
Umsetzung von Kinderrechten (z.B. Mitbestimmung, Beschwerdemöglichkeiten)	☐	☐	☐	☐	☐	☐
Vorschule	☐	☐	☐	☐	☐	☐
Zusammenarbeit mit Familien/Erziehungspartnerschaft	☐	☐	☐	☐	☐	☐

36 Wie viele Kinder haben <u>derzeit</u> pandemiebedingt einen erhöhten Förderungsbedarf im Vergleich zum Regelbetrieb vor dem Lockdown in Bezug auf ...

☞ *Bitte machen Sie in jeder Zeile eine Angabe.*

	Viel weniger Kinder 1	2	3	4	Viel mehr Kinder 5
Sprachliche Bildung	☐	☐	☐	☐	☐
MINT (Mathematik, Informatik, Naturwissenschaften, Technik)	☐	☐	☐	☐	☐
Förderung von Motorik/Bewegung	☐	☐	☐	☐	☐
Musikalisch-künstlerische Bildung	☐	☐	☐	☐	☐
Kompetenzförderung im Umgang mit digitalen Medien	☐	☐	☐	☐	☐
Gesundheitsförderung	☐	☐	☐	☐	☐
Förderung der sozial-emotionalen Entwicklung	☐	☐	☐	☐	☐

37 Wurden in Ihrer Einrichtung <u>während des Lockdowns</u> zu jeder Zeit alle Kinder betreut, die normalerweise im Regelbetrieb anwesend sind (vor Corona)?

Ja 1 ☐ → ***Bitte weiter mit Frage 39***

Nein 0 ☐ → ***Bitte weiter mit Frage 38***

38 Inwiefern wurden <u>während des Lockdowns</u> folgende Aktionen für Kinder durchgeführt, die in dieser Zeit nicht in der Einrichtung betreut wurden?

☞ *Bitte machen Sie in jeder Zeile eine Angabe.*

	Gar nicht 1	Manchmal 2	Oft 3
Briefe/E-Mails an Kinder	☐	☐	☐
Bereitstellung von Bastelmaterial zur Abholung	☐	☐	☐
Verschicken von Bastelmaterial	☐	☐	☐
Nutzung des öffentlichen Raums für Aktionen (z.B. Steinschlange)	☐	☐	☐
Digitaler Morgenkreis	☐	☐	☐
Andere digitale Angebote	☐	☐	☐

39 Werden in Ihrer Einrichtung <u>derzeit</u> alle angemeldeten Kinder betreut, die normalerweise im Regelbetrieb anwesend sind?

Ja 1 ☐ → ***Bitte weiter mit Frage 41***

Nein 0 ☐ → ***Bitte weiter mit Frage 40***

40 Inwieweit werden folgende Aktionen von Ihrer Einrichtung <u>derzeit</u> für Kinder durchgeführt, die nicht in der Einrichtung betreut werden?

☞ *Bitte machen Sie in jeder Zeile eine Angabe.*

	Gar nicht 1	Manchmal 2	Oft 3
Briefe/E-Mails an Kinder	☐	☐	☐
Bereitstellung von Bastelmaterial zur Abholung	☐	☐	☐
Verschicken von Bastelmaterial	☐	☐	☐
Nutzung des öffentlichen Raums für Aktionen (z.B. Steinschlange)	☐	☐	☐
Digitaler Morgenkreis	☐	☐	☐
Andere digitale Angebote	☐	☐	☐

41 **Was waren/sind für Ihre Einrichtung die größten pandemiebedingten organisatorischen/pädagogischen/hygienischen Herausforderungen?**

42 **Mit welchen Lösungen sind Sie in Ihrer Einrichtung diesen Herausforderungen begegnet?**

43 **Welches Datum haben wir heute?**

☐ Tag ☐ Monat ☐ Jahr

44 **Haben Sie den Fragebogen am Stück ausgefüllt?**

Ja 1 ☐

Nein mit einer/mehreren Unterbrechungen 2 ☐

Herzlichen Dank für Ihre Mithilfe.

Bitte schicken Sie den ausgefüllten Fragebogen
im portofreien Umschlag an infas zurück.

7370/2020
