# Supplementary material for: Establishing multi-perspective instruments in early education during COVID-19: measuring the implementation of protective measures and the subjective level of information about pandemic-related regulations
Source: Meas Instrum Soc Sci. 2022 May 12;4(1):7. doi: 10.1186/s42409-022-00033-2 (PMC9096761; doi:10.1186/s42409-022-00033-2)
Supplement: Supplementary file 4 — Additional file 4. ERiK directors‘ questionnaire [file 42409_2022_33_MOESM4_ESM.pdf]

# Fragebogen

# für die Einrichtungsleitung

**Sehr geehrte Einrichtungsleitung,**

im Auftrag des Deutschen Jugendinstituts (DJI) führt das infas Institut für angewandte Sozialwissenschaft eine wissenschaftliche Befragung von Einrichtungsleitungen und pädagogischem Personal in Kindertageseinrichtungen durch. Diese Befragung ist Teil einer Studie zur Entwicklung von Rahmenbedingungen in der Kindertagesbetreuung (kurz ERiK). Ihre Einrichtung wurde nach einem statistischen Zufallsverfahren ausgewählt.

In diesem Fragebogen interessieren wir uns für Ihre Einschätzung als **Leitung dieser Einrichtung**. Ihre Unterstützung ist für den Erfolg der Studie dabei von größter Bedeutung. Ihre Angaben werden nur in anonymisierter Form, das heißt ohne Namen und Adresse, und nur zusammengefasst mit den Angaben der anderen Befragten ausgewertet. Alle Regeln des Datenschutzes werden vollständig eingehalten.

**Am einfachsten können Sie den Fragebogen online ausfüllen.**

Bitte geben Sie hierzu folgende Adresse in Ihrem Internetbrowser ein:

Ihr persönlicher Zugangscode lautet:

Alternativ zu dem Online-Fragebogen können Sie diesen schriftlichen Fragebogen ausfüllen und in dem beigefügten portofreien Rückumschlag an infas zurücksenden.

**Wie ist der Fragebogen auszufüllen?**

Kreuzen Sie bitte die jeweils zutreffenden Antwortmöglichkeiten in den dafür vorgesehenen Kästchen an: ☒

In die großen Kästchen setzen Sie bitte die jeweils erfragten Zahlen oder Angaben ein: 87

Bitte achten Sie auf entsprechende Hinweise zum Ausfüllen von Fragen: ☞ *Bitte machen Sie nur eine Angabe.*

Bitte achten Sie auf entsprechende Hinweise zum Überspringen von Fragen: → ***Bitte weiter mit Frage xy***

DJI Deutsches Jugendinstitut

infas

infas Institut für angewandte Sozialwissenschaft GmbH

Postfach 240101
53154 Bonn
Tel. 0800/73 84 500
erik@infas.de

7135/2020/L

## 1 In welcher Funktion arbeiten Sie in der Kindertageseinrichtung?

*☞ Bitte machen Sie nur eine Angabe.*

- Leitung 1 ☐
- Stellvertretende Leitung 2 ☐
- Teil eines Leitungsteams 3 ☐
- Kommissarische Leitung 4 ☐
- Sonstige Leitungsfunktion, und zwar: 5 ☐

*☞ Bitte angeben:*

## 2 Wie ist die Leitung in Ihrer Kindertageseinrichtung formal geregelt?

*☞ Bitte machen Sie nur eine Angabe.*

- Die Leitung wird durch eine Person übernommen. 1 ☐
- Die Leitung wird durch ein Leitungstandem, bestehend aus zwei gleichberechtigten Personen, übernommen. 2 ☐
- Die Leitung wird durch eine Leitung und eine Stellvertretung übernommen. 3 ☐
- Eine Leitung ist für mehrere Einrichtungen zuständig. 4 ☐
- Es gibt keine vertraglich festgelegte Leitung. 5 ☐

## 3 Gibt es in Ihrer Kindertageseinrichtung eine verbindliche Arbeitsplatzbeschreibung für Leitungskräfte?

- Ja 1 ☐
- Nein 2 ☐

## 4 Kommen wir nun zu Ihren Leitungsaufgaben (pädagogische Leitung und Verwaltungsaufgaben). Wie viele Stunden pro Woche …

**… sind vertraglich für Leitungsaufgaben festgelegt?**

Stunden

**… fallen tatsächlich für Leitungsaufgaben an?**

Stunden

## 5 Bitte geben Sie die Art des Trägers Ihrer Kindertageseinrichtung an.

- Öffentlicher Träger (Stadt, Gemeinde) 1 ☐
- Frei-gemeinnütziger, nicht-konfessioneller Träger (z.B. Arbeiterwohlfahrt) 2 ☐
- Frei-gemeinnütziger, konfessioneller Träger (z.B. Caritasverband, Diakonie) 3 ☐
- Privat-gewerblicher Träger 4 ☐
- Sonstiger gemeinnütziger Träger 5 ☐
- Sonstiger Träger 6 ☐

## 6 Ist der Träger eine Elterninitiative oder ein Unternehmens-/Betriebsteil (Betriebskindertagesstätte)?

- Eine Elterninitiative 1 ☐
- Ein Unternehmens-/Betriebsteil 2 ☐
- Keines von beiden 3 ☐

## 7 Hat der Träger neben Ihrer Einrichtung noch andere Einrichtungen, in denen Kinder von 0 bis 6 Jahren betreut werden?

| | | |
|---|---|---|
| Ja | 1 | ☐ |
| Nein | 2 | ☐ |
| Weiß nicht | 8 | ☐ |

## 8 Ist die Kindertageseinrichtung an den folgenden Bundesprogrammen beteiligt?

*Bitte machen Sie in jeder Zeile eine Angabe.*

| | 1 Ja | 2 Nein |
|---|---|---|
| BiSS: Bildung durch Sprache und Schrift | ☐ | ☐ |
| Fachkräfteoffensive ErzieherInnen | ☐ | ☐ |
| Kita-Einstieg: Brücken bauen in frühe Bildung | ☐ | ☐ |
| Quereinstieg - Männer und Frauen in Kitas | ☐ | ☐ |
| Sprach-Kitas: Weil Sprache der Schlüssel zur Welt ist | ☐ | ☐ |
| ProKindertagespflege: Wo Bildung für die Kleinsten beginnt | ☐ | ☐ |
| Sonstiges | ☐ | ☐ |

## 9 An welchen Landesprogrammen ist die Kindertageseinrichtung beteiligt?

*Bitte angeben:*

Unsere Einrichtung ist an keinem Landesprogramm beteiligt ☐

## 10 Wie viele MitarbeiterInnen sind insgesamt in Ihrer Kindertageseinrichtung tätig?

MitarbeiterInnen in Ihrer Einrichtung insgesamt

## 11 Wie viele der MitarbeiterInnen in Ihrer Kindertageseinrichtung sind tätig als...?

*Bitte zählen Sie alle Personen nur einmal. Bitte zählen Sie auch alle MitarbeiterInnen mit, die sich aktuell in Elternzeit oder im Krankenstand befinden. Zählen Sie nicht diejenigen MitarbeiterInnen, die sich ausschließlich um Hortkinder kümmern.*

| | Anzahl | Keine |
|---|---|---|
| Einrichtungsleitung | | ☐ |
| Pädagogische Fachkraft (einschließlich HeilpädagogInnen) | | ☐ |
| Assistenzkraft | | ☐ |
| Förderkraft (für Kinder, die nach SGB VIII oder nach SGB XII Eingliederungshilfe erhalten) | | ☐ |
| Sonstige Förderkräfte (z.B. für die Bereiche Sprache, Sport, Musik) | | ☐ |
| Verwaltungskraft | | ☐ |
| Angehende pädagogische Fachkräfte im Praktikum (z.B. im Anerkennungsjahr) | | ☐ |
| PraktikantInnen (ohne angehende pädagogische Fachkräfte) | | ☐ |
| Person im Freiwilligen sozialen Jahr/Bundesfreiwilligendienst (FSJ/FÖJ/BFD) | | ☐ |
| Sonstige | | ☐ |

## 12 Wie viele Ihrer pädagogischen MitarbeiterInnen sind zusätzlich qualifiziert als ... ?

| | Anzahl | Keine | Weiß nicht |
|---|---|---|---|
| Interkulturelle Fachkraft (IKF) | ☐ | ☐ | ☐ |
| Fachkraft für Inklusion | ☐ | ☐ | ☐ |

## 13 Wie viele Planstellen für pädagogische MitarbeiterInnen stehen Ihnen in Ihrer Kindertageseinrichtung zur Verfügung?

☞ *Beispiel: 2,5 Planstellen (für zwei Planstellen und eine halbe)*

☐ Anzahl der Planstellen

## 14 Wie viele Männer sind als pädagogisches Personal in Ihrer Kindertageseinrichtung tätig?

☐ Anzahl der Männer als pädagogisches Personal

☞ *Wenn Sie als ausfüllende Leitung männlich sind, zählen Sie sich bitte dazu.*

davon als Leitung ☐

## 15 Wie viele Stunden stehen dem pädagogischen Personal (pro Stelle) wöchentlich an mittelbarer pädagogischer Arbeitszeit zu? (bei einer Vollzeitstelle)

☞ *Mit mittelbarer pädagogischer Arbeitszeit sind etwa Vor- und Nachbereitungszeiten, Zusammenarbeit mit Eltern und KooperationspartnerInnen, Teamgespräche etc. gemeint.*

| | Stunden | Keine |
|---|---|---|
| GruppenleiterIn (falls vorhanden) | ☐ | ☐ |
| Pädagogische Fachkraft (einschließlich HeilpädagogInnen) | ☐ | ☐ |
| Assistenzkraft | ☐ | ☐ |
| Förderkraft (für Kinder, die nach SGB VIII oder nach SGB XII Eingliederungshilfe erhalten) | ☐ | ☐ |

## 16 In welchen der folgenden Modelle der Ausbildung befinden sich Ihre angehenden pädagogischen Fachkräfte?

☞ *Bitte machen Sie in jeder Zeile eine Angabe.*

| | 1 Ja | 2 Nein |
|---|---|---|
| Berufsfachschulische Ausbildung für Kinderpflege und/oder Sozialassistenz | ☐ | ☐ |
| Regelausbildung: Fachschulische Ausbildung zur Erzieherin oder zum Erzieher (z.B. Fachakademie) | ☐ | ☐ |
| Verkürztes Ausbildungsmodell (integrativ): Fachschulische Ausbildung zur Erzieherin oder zum Erzieher (z.B. 2-jährig) | ☐ | ☐ |
| Praxisintegrierte Ausbildung an einer Fachschule und in der Praxis (z.B. PIA oder OptiPrax) | ☐ | ☐ |
| Studium der Kindheitspädagogik an einer Hochschule | ☐ | ☐ |

Wir haben keine angehenden pädagogischen Fachkräfte. ☐



7135/2020

**17** **Inwiefern wird Ihre pädagogische Arbeit aktuell durch folgende Umstände beeinträchtigt?**

*☞ Bitte machen Sie in jeder Zeile eine Angabe.*

| | Gar nicht beein-trächtigt 1 | 2 | 3 | 4 | 5 | Stark beein-trächtigt 6 |
|---|---|---|---|---|---|---|
| Mangel an pädagogischen Fachkräften mit besonderen Kompetenzen in der Arbeit mit Kindern mit benachteiligtem sozio-ökonomischem Hintergrund | ☐ | ☐ | ☐ | ☐ | ☐ | ☐ |
| Mangel an pädagogischen Fachkräften mit besonderen Kompetenzen zur kultursensiblen Pädagogik | ☐ | ☐ | ☐ | ☐ | ☐ | ☐ |
| Mangel an pädagogischen Fachkräften mit besonderen Kompetenzen im Bereich der Inklusion | ☐ | ☐ | ☐ | ☐ | ☐ | ☐ |

**18** **Gibt es in Ihrer Kindertageseinrichtung Stellen für pädagogische Fachkräfte, die aufgrund mangelnder Bewerbungen bereits 6 Monate oder länger nicht besetzt werden konnten?**

- Ja 1 ☐
- Nein 2 ☐
- Weiß nicht 8 ☐

**19** **Wie viele MitarbeiterInnen haben Ihre Einrichtung in den letzten 12 Monaten verlassen (z.B. aufgrund von Befristungen, Kündigungen etc.)?**

☐ Anzahl MitarbeiterInnen

Keine ☐ → *Bitte weiter mit Frage 21*

**20** **Welche der im Folgenden aufgeführten Gründe treffen auf die MitarbeiterInnen zu, die Ihre Kindertageseinrichtung in den letzten 12 Monaten verlassen haben?**

*☞ Bitte machen Sie in jeder Zeile eine Angabe.*

| | 1 Ja | 2 Nein |
|---|---|---|
| Befristungen/Auslaufende Verträge | ☐ | ☐ |
| Kündigung aufgrund einer anderen Arbeitsstelle in einer Kindertageseinrichtung | ☐ | ☐ |
| Kündigung für die Position einer Leitung in einer Kindertageseinrichtung | ☐ | ☐ |
| Kündigung aufgrund einer anderen Arbeitsstelle im System der frühkindlichen Bildung (z.B. Fachberatung) | ☐ | ☐ |
| Kündigung aufgrund einer anderen Arbeitsstelle | ☐ | ☐ |
| Kündigung aufgrund einer beruflichen Umorientierung | ☐ | ☐ |
| Kündigung auf Wunsch der Einrichtung | ☐ | ☐ |
| Rente | ☐ | ☐ |
| Private Gründe | ☐ | ☐ |
| Sonstiges | ☐ | ☐ |

**21** **Wie viele MitarbeiterInnen stehen Ihrer Einrichtung aktuell aufgrund längerer Abwesenheit (mind. 6 Wochen) nicht zur Verfügung (Krankheit, Elternzeit etc.)?**

Anzahl MitarbeiterInnen ☐ Keine ☐

**22** **An wie vielen Tagen kam es in den letzten 6 Monaten vor, dass der vorgegebene Personal-schlüssel in Ihrer Kindertageseinrichtung nicht eingehalten werden konnte?**

Ungefähre Anzahl der Tage ☐ → *Bitte weiter mit Frage 23*

Das ist in unserer Einrichtung nicht vorgekommen. ☐ → *Bitte weiter mit Frage 24*

## 23 Warum konnte der Personalschlüssel nicht eingehalten werden?

*☞ Bitte machen Sie in jeder Zeile eine Angabe.*

| | Ja [1] | Nein [2] |
|---|---|---|
| Krankheit, Beschäftigungsverbot bei Schwangerschaft, Mutterschutz | ☐ | ☐ |
| Unbesetzte Stellen | ☐ | ☐ |
| Fort- und Weiterbildungen | ☐ | ☐ |
| Urlaub | ☐ | ☐ |

## 24 Kam es in den letzten 6 Monaten in Ihrer Einrichtung vor, dass Sie Personalausfälle ausgleichen mussten?

Ja 1 ☐ → ***Bitte weiter mit Frage 25***

Nein 2 ☐ → ***Bitte weiter mit Frage 26***

## 25 Wie haben Sie diese Personalausfälle ausgeglichen?

*☞ Bitte machen Sie in jeder Zeile eine Angabe.*

| | Ja [1] | Nein [2] |
|---|---|---|
| Durch Einsatz von Springerkräften | ☐ | ☐ |
| Durch bezahlte Stundenaufstockung von Teilzeitkräften | ☐ | ☐ |
| Durch Übernahme der pädagogischen Arbeit durch die Leitung | ☐ | ☐ |
| Durch Einsatz von pädagogischem Personal aus Zeitarbeitsfirmen oder freiberuflichen ErzieherInnen | ☐ | ☐ |
| Durch Einsatz von einer/mehreren Tagespflegeperson/en | ☐ | ☐ |
| Durch Überstunden des pädagogischen Personals | ☐ | ☐ |
| Durch Mobilisierung von ehrenamtlichen Kräften/Eltern | ☐ | ☐ |
| Durch Zusammenlegung der Gruppen | ☐ | ☐ |
| Durch vorübergehende Schließung | ☐ | ☐ |
| Durch Kürzung der Öffnungszeiten | ☐ | ☐ |

## 26 Gibt es ein schriftliches Einarbeitungskonzept für neue pädagogische MitarbeiterInnen?

*☞ Bei dieser Frage geht es nicht um die Einarbeitung der Einrichtungsleitung oder der PraktikantInnen.*

Ja 1 ☐

Nein 2 ☐

## 27 Wer übernimmt die Einarbeitung neuer pädagogischer MitarbeiterInnen in Ihrer Einrichtung?

*☞ Bitte machen Sie in jeder Zeile eine Angabe. Bei dieser Frage geht es nicht um die Einarbeitung der Einrichtungsleitung oder der PraktikantInnen.*

| | Ja [1] | Nein [2] |
|---|---|---|
| Leitung | ☐ | ☐ |
| Stellvertretende Leitung | ☐ | ☐ |
| Eine dafür bestimmte pädagogische Fachkraft | ☐ | ☐ |
| Team | ☐ | ☐ |

## 28 Welche der im Folgenden aufgeführten verbindlichen Personalentwicklungs-/Personalbindungsmaßnahmen gibt es in Ihrer Einrichtung?

☞ *Bitte machen Sie in jeder Zeile eine Angabe.*

| | Ja [1] | Nein [2] |
|---|---|---|
| Vereinbarung konkreter Fort- und Weiterbildungsmaßnahmen | ☐ | ☐ |
| Besprechen von Zielen und Maßnahmen der individuellen professionellen Weiterentwicklung | ☐ | ☐ |
| Beurteilung der festgelegten Ziele | ☐ | ☐ |
| Supervision | ☐ | ☐ |
| Hospitation in anderen Einrichtungen | ☐ | ☐ |
| Angebote zur Gesundheitsförderung | ☐ | ☐ |
| Spezifische Maßnahmen zur Verbesserung der Arbeitsbedingungen älterer MitarbeiterInnen | ☐ | ☐ |
| Flexible Arbeitszeiten (soweit im Rahmen der Öffnungszeiten möglich) | ☐ | ☐ |
| Team-/Klausurtage | ☐ | ☐ |
| Befragung zur Mitarbeiterzufriedenheit | ☐ | ☐ |

## 29 Im Folgenden geht es um die Plätze und Kinder in Ihrer Kindertageseinrichtung. Wie viele genehmigte Plätze gemäß Betriebserlaubnis hat Ihre Einrichtung?

☐ Anzahl genehmigte Plätze

## 30 Gab es in Ihrer Einrichtung zum Stichtag 01.03.2020 freie Plätze?

Ja 1 ☐ → *Bitte weiter mit Frage 31*

Nein 2 ☐ → *Bitte weiter mit Frage 32*

## 31 Wie viele freie Plätze hatten Sie zum Stichtag 01.03.2020 in Ihrer Kindertageseinrichtung in den aufgeführten Altersgruppen?

| | Anzahl | | |
|---|---|---|---|
| Anzahl freie Plätze für unter 3-Jährige | ☐ | Keine | ☐ |
| Anzahl freie Plätze für 3-Jährige bis Schuleintritt | ☐ | Keine | ☐ |
| Anzahl freie Plätze für Grundschulkinder | ☐ | Keine | ☐ |

## 32 Entspricht das Angebot von Betreuungsplätzen für Kinder in Ihrer Kindertageseinrichtung der Nachfrage?

☞ *Bitte machen Sie in jeder Zeile eine Angabe.*

| | Zu geringes Angebot [1] | Ausreichend [2] | Zu großes Angebot [3] | Trifft nicht zu [98] |
|---|---|---|---|---|
| Platzangebot für unter 3-Jährige | ☐ | ☐ | ☐ | ☐ |
| Platzangebot für 3-Jährige bis Schuleintritt | ☐ | ☐ | ☐ | ☐ |
| Platzangebot für Grundschulkinder | ☐ | ☐ | ☐ | ☐ |

## 33 Wer entscheidet über die Vergabe von Plätzen in Ihrer Einrichtung?

☞ *Bitte wählen Sie die Institution aus, welche im Zweifelsfall entscheidet.*

Die Einrichtung 1 ☐

Der Träger 2 ☐

Das Jugendamt 3 ☐

**34** **Welche Kriterien werden bei der Vergabe von Kinderbetreuungsplätzen berücksichtigt?**

☞ *Bitte machen Sie in jeder Zeile eine Angabe.*

| | 1 Ja | 2 Nein | Weiß nicht |
|---|---|---|---|
| Kinder mit einer persönlichen Notlage | ☐ | ☐ | ☐ |
| Kinder mit einem alleinerziehenden Elternteil | ☐ | ☐ | ☐ |
| Wartezeit eines Kindes seit der Anmeldung | ☐ | ☐ | ☐ |
| Beschäftigungsstatus der Erziehungsberechtigten | ☐ | ☐ | ☐ |
| Geschwisterkinder in der Einrichtung | ☐ | ☐ | ☐ |
| Wohnortnähe zur Einrichtung | ☐ | ☐ | ☐ |
| Angaben der Eltern bezüglich ihrer Wunschkita | ☐ | ☐ | ☐ |
| Besonderes Engagement der Eltern bzw. Erziehungsberechtigten | ☐ | ☐ | ☐ |
| Konfession/Religionszugehörigkeit des Kindes | ☐ | ☐ | ☐ |
| Gewünschter Betreuungsumfang | ☐ | ☐ | ☐ |
| Alter des Kindes | ☐ | ☐ | ☐ |
| Förderbedarf | ☐ | ☐ | ☐ |
| Betriebszugehörigkeit der Eltern | ☐ | ☐ | ☐ |

**35** **Mit welchem Gruppenkonzept arbeiten Sie in Ihrer Kindertageseinrichtung?**

| | |
|---|---|
| Offen | 1 ☐ |
| Teilweise offen | 2 ☐ |
| Feste Gruppenstruktur | 3 ☐ |

**36** **Wie viele Kinder wurden zum Stichtag 01.03.2020 in Ihrer Kindertageseinrichtung betreut?**

| | Anzahl | Keine |
|---|---|---|
| **Kinder insgesamt unter 3 Jahren:** | | ☐ |
| Davon: Kinder mit besonderen Förderbedarfen (SGB VIII oder nach SGB XII/Eingliederungshilfe) | | ☐ |
| Davon: Kinder mit nicht deutscher Familiensprache (nicht: mehrsprachige Kinder mit deutscher Sprache einer Bezugsperson) | | ☐ |
| Davon: Kinder, die einen Ganztagsplatz haben | | ☐ |
| **Kinder insgesamt ab 3 Jahren bis zum Schuleintritt:** | | ☐ |
| Davon: Kinder mit besonderen Förderbedarfen (SGB VIII oder nach SGB XII/Eingliederungshilfe) | | ☐ |
| Davon: Kinder mit nicht deutscher Familiensprache (nicht: mehrsprachige Kinder mit deutscher Sprache einer Bezugsperson) | | ☐ |
| Davon: Kinder, die einen Ganztagsplatz haben | | ☐ |
| **Grundschulkinder insgesamt:** | | ☐ |



7135/2020

## 37 Schätzen Sie bitte den Prozentsatz der Kinder in Ihrer Kindertageseinrichtung, welche die folgenden persönlichen Merkmale aufweisen.

*Bitte machen Sie in jeder Zeile eine Angabe.*

| | Keine 1 | 1 bis 10 % 2 | 11 bis 30 % 3 | 31 bis 60 % 4 | über 60 % 5 | Weiß nicht 8 |
|---|---|---|---|---|---|---|
| Kinder mit sozio-ökonomisch benachteiligtem Hintergrund | ☐ | ☐ | ☐ | ☐ | ☐ | ☐ |
| Kinder mit Fluchthintergrund | ☐ | ☐ | ☐ | ☐ | ☐ | ☐ |

## 38 Wie viele von den Kindern mit Förderbedarf erhalten in Ihrer Einrichtung Eingliederungshilfe nach SGB VIII/SGB XII wegen...

**...körperlicher Behinderung?**

Anzahl der Kinder mit körperlicher Behinderung ☐ Keine ☐

**...geistiger Behinderung?**

Anzahl der Kinder mit geistiger Behinderung ☐ Keine ☐

**...drohender oder seelischer Behinderung (u. a. auch wegen Entwicklungsverzögerung)?**

Anzahl der Kinder mit drohender oder seelischer Behinderung ☐ Keine ☐

## 39 Wie viele Kinder mit diagnostizierten Sprach-, Verhaltens- oder Entwicklungsstörungen waren zum Stichtag 01.03.2020 in Ihrer Einrichtung?

*Bitte machen Sie in jeder Zeile eine Angabe.*

| | 2 Nein | 1 Ja | | Anzahl der Kinder |
|---|---|---|---|---|
| Kinder mit diagnostizierten Sprachstörungen (nicht gemeint sind Stottern/Lispeln) | ☐ | ☐ | → | ☐ |
| Kinder mit diagnostizierten Verhaltensstörungen (z.B. Störungen des Sozialverhaltens) | ☐ | ☐ | → | ☐ |
| Kinder mit anderen diagnostizierten Entwicklungsstörungen (z.B. motorische Störungen) | ☐ | ☐ | → | ☐ |

## 40 Werden in Ihrer Einrichtung bestimmte Formen der Sprachförderung eingesetzt?

*Bitte machen Sie in jeder Zeile eine Angabe.*

| | Nein 1 | Ja, in der Gesamt-gruppe 2 | Ja, in der Klein-gruppe 3 | Ja, als Einzel-förderung 4 |
|---|---|---|---|---|
| Vorstrukturierte Förderprogramme mit vorgegebenen Lerneinheiten (z.B. „Kon-Lab“ oder „Hören, Lauschen, Lernen“) | ☐ | ☐ | ☐ | ☐ |
| Gezielte Vorleseaktivitäten | ☐ | ☐ | ☐ | ☐ |
| Gezielte Sprachspiele | ☐ | ☐ | ☐ | ☐ |

## 41 Wie findet die Beobachtung und Dokumentation der Sprachkompetenz bei Kindern in Ihrer Einrichtung statt?

*Bitte machen Sie in jeder Zeile eine Angabe.*

| | Ja (1) | Nein (2) | Weiß nicht (8) |
|---|---|---|---|
| Durch freie Beobachtung der Kinder | ☐ | ☐ | ☐ |
| Durch standardisierte Beobachtungsbogen (z.B. Seldak, Sismik) | ☐ | ☐ | ☐ |
| Durch standardisierte Tests (z.B. HASE-Screening) | ☐ | ☐ | ☐ |
| Abstimmung mit den Ergebnissen der kinderärztlichen U-Untersuchungen | ☐ | ☐ | ☐ |
| Sonstiges, und zwar | ☐ | ☐ | ☐ |

*Bitte angeben:*

[ ]

## 42 Gibt es in Ihrer Kindertageseinrichtung Fördermaßnahmen für Kinder mit besonderen Bedarfen?

*Bitte machen Sie in jeder Zeile eine Angabe.*

| | Ja (1) | Nein (2) | Weiß nicht (8) |
|---|---|---|---|
| Kinder mit Entwicklungsverzögerungen | ☐ | ☐ | ☐ |
| Kinder mit (drohenden) Behinderungen (Gemeint sind Kinder, die gemäß § 39, 40 BSHG oder § 35a KJHG behindert oder von Behinderung bedroht sind und für die ein besonderer Anspruch auf Eingliederungshilfe besteht.) | ☐ | ☐ | ☐ |
| Kinder, die eine besonders schnelle Entwicklung aufweisen | ☐ | ☐ | ☐ |
| Kinder mit einer chronischen Erkrankung | ☐ | ☐ | ☐ |
| Kinder mit herausfordernden Verhaltensweisen | ☐ | ☐ | ☐ |

## 43 Nennen Sie bitte die Öffnungszeiten Ihrer Kindertageseinrichtung.

*Nennen Sie bitte die Tage und Zeiten, an denen Ihre Einrichtung geöffnet oder geschlossen ist. Bitte machen Sie für jeden Wochentag eine Angabe.*

| | | Ab welcher Uhrzeit? | | Bis zu welcher Uhrzeit? | Geschlossen |
|---|---|---|---|---|---|
| **Montag** | von | : | bis | : | ☐ |
| **Dienstag** | von | : | bis | : | ☐ |
| **Mittwoch** | von | : | bis | : | ☐ |
| **Donnerstag** | von | : | bis | : | ☐ |
| **Freitag** | von | : | bis | : | ☐ |
| **Samstag** | von | : | bis | : | ☐ |
| **Sonntag** | von | : | bis | : | ☐ |

**44** **Wird in Ihrer Kindertageseinrichtung in Ausnahmefällen eine Betreuung außerhalb der genannten Zeiten angeboten?**

Ja 1 ☐
Nein 2 ☐

**45** **An wie vielen Tagen war Ihre Einrichtung wegen Urlaub, Ferien oder Weiterbildungen in den vergangenen 12 Monaten insgesamt geschlossen?**

*☞ Bitte zählen Sie diejenigen Tage nicht mit, an denen die Einrichtung ohnehin geschlossen ist.*

☐ Anzahl Tage in den vergangenen 12 Monaten insgesamt

**46** **Welche vertraglich vereinbarte Betreuungszeit in Stunden pro Woche bieten Sie für Kinder unter 3 Jahren an?**

*☞ Bitte machen Sie in jeder Zeile eine Angabe.*

| | 1 Ja | 2 Nein |
|---|---|---|
| Bis zu 25 Stunden | ☐ | ☐ |
| 26 bis unter 36 Stunden | ☐ | ☐ |
| 36 bis unter 40 Stunden | ☐ | ☐ |
| 40 bis unter 45 Stunden | ☐ | ☐ |
| 45 Stunden und mehr | ☐ | ☐ |

**47** **Welche vertraglich vereinbarte Betreuungszeit in Stunden pro Woche bieten Sie für Kinder ab 3 Jahren bis zum Alter des Schuleintritts an?**

*☞ Bitte machen Sie in jeder Zeile eine Angabe.*

| | 1 Ja | 2 Nein |
|---|---|---|
| Bis zu 25 Stunden | ☐ | ☐ |
| 26 bis unter 36 Stunden | ☐ | ☐ |
| 36 bis unter 40 Stunden | ☐ | ☐ |
| 40 bis unter 45 Stunden | ☐ | ☐ |
| 45 Stunden und mehr | ☐ | ☐ |

**48** **Gibt es für Ihre Einrichtung ein schriftlich festgelegtes pädagogisches Konzept?**

Ja 1 ☐
Nein 2 ☐
Weiß nicht 8 ☐

**49** **Wie viele Sprachen spricht das pädagogische Personal in Ihrer Einrichtung - neben Deutsch - mit den Kindern?**

Nur Deutsch 1 ☐
Deutsch und eine weitere Sprache 2 ☐
Deutsch und zwei weitere Sprachen 3 ☐
Deutsch und drei weitere Sprachen 4 ☐
Deutsch und mehr als drei weitere Sprachen 5 ☐
Weiß nicht 8 ☐

## 50 Welche der im Folgenden aufgeführten Varianten der Fachberatung nutzen Sie in Ihrer Kindertageseinrichtung?

*Bitte machen Sie in jeder Zeile eine Angabe.*

| | 1 Ja | 2 Nein |
|---|---|---|
| Fachberatung des Trägers bzw. Trägerverbands | ☐ | ☐ |
| Freiberufliche Fachberatung | ☐ | ☐ |
| Fachberatung durch das Jugendamt/die Kommune | ☐ | ☐ |
| Sonstige Fachberatung | ☐ | ☐ |
| Für meine Einrichtung ist keine zuständige Fachberatung vorhanden | ☐ | ☐ |

## 51 Wie oft hatten Sie in den letzten 12 Monaten Kontakt zu einer Fachberatung (auch telefonisch)?

| | |
|---|---|
| Gar nicht | 1 ☐ |
| Einmal im Jahr | 2 ☐ |
| Mehrmals im Jahr | 3 ☐ |
| Mindestens einmal monatlich | 4 ☐ |
| Mindestens einmal wöchentlich | 5 ☐ |

## 52 Welche Maßnahmen zur Qualitätssicherung führen Sie in Ihrer Kindertageseinrichtung durch?

*Definition: Bei der **internen** Evaluation handelt es sich um Formen der Selbstevaluation. Das heißt, die Kita-Leitung und das pädagogische Personal reflektieren und bewerten mithilfe eines strukturierten Verfahrens ihre eigene Arbeit sowie die Arbeit des Kita-Teams.*

*Definition: Bei der **externen** Evaluation handelt es sich um eine Fremdevaluation, die von externen ExpertInnen durchgeführt wird. Diese bewerten nach einem ausgewählten Vorgehen die Arbeit der Kitas, z.B. über Beobachtungen des pädagogischen Alltags, Gespräche mit Leitungen und pädagogischem Personal oder die Befragung von Eltern.*

*Bitte machen Sie in jeder Zeile eine Angabe.*

| | 1 Ja | 2 Nein |
|---|---|---|
| Regelmäßige Durchführung von externen Evaluationen (mindestens alle 5 Jahre) | ☐ | ☐ |
| Regelmäßige Durchführung von internen Evaluationen (mindestens alle 3 Jahre) | ☐ | ☐ |
| Regelmäßige Inspektion vor Ort durch das Jugendamt | ☐ | ☐ |
| Verpflichtende Weiterbildungsangebote für pädagogisches Personal | ☐ | ☐ |
| Regelmäßige Inanspruchnahme der Fachberatung | ☐ | ☐ |
| Einbezug von Eltern in die Qualitätserfassung/-entwicklung | ☐ | ☐ |
| Einbezug von Kindern in die Qualitätserfassung/-entwicklung | ☐ | ☐ |
| Andere Formen der Überprüfung, und zwar | ☐ | ☐ |

*Bitte angeben:*

**53** **Hat die von Ihnen geleitete Einrichtung in den letzten 12 Monaten an einer oder mehreren der folgenden Qualitätsentwicklungsmaßnahmen teilgenommen?**

☞ *Bitte machen Sie in jeder Zeile eine Angabe.*

| | Ja | Nein |
|---|---|---|
| Das Paritätische Qualitätssystem (PQS Sys) | ☐ | ☐ |
| Integrierte Qualitäts- und Personalentwicklung (IQUE) | ☐ | ☐ |
| Kindergarteneinschätzskala (KES-R) | ☐ | ☐ |
| KLAX gGmbH | ☐ | ☐ |
| Qualitätsmanagement in katholischen Kindertageseinrichtungen (KTK Gütesiegel) | ☐ | ☐ |
| Lernorientierte Qualitätssteigerung für Kindergärten (LQK) | ☐ | ☐ |
| Nationales Gütesiegel nach PädQUIS | ☐ | ☐ |
| Qualität im Situationsansatz (QUASI) | ☐ | ☐ |
| Evangelisches Gütesiegel BETA | ☐ | ☐ |
| Qualitätsmanagement in Kindertageseinrichtungen der Arbeiterwohlfahrt (AWO-QM) | ☐ | ☐ |
| Träger zeigen Profil (TQ) | ☐ | ☐ |
| Andere Qualitätsentwicklungsmaßnahmen, und zwar: | ☐ | ☐ |

☞ *Bitte angeben:*

☐

**Im Folgenden geht es um Fragen zu Ihrer Beschäftigung in der Kindertageseinrichtung.**

**54** **Wie ist Ihr Beschäftigungsstatus?**

☞ *Bitte geben Sie den Beschäftigungsstatus an, den Sie in dieser Einrichtung haben.*

| | |
|---|---|
| Unbefristetes Angestelltenverhältnis | 1 ☐ |
| Befristetes Angestelltenverhältnis | 2 ☐ |
| Selbständig | 3 ☐ |

**55** **Wie viele Stunden pro Woche beträgt Ihre ...**

**... vertraglich festgelegte Arbeitszeit?**

☐ Stunden

**... tatsächliche Arbeitszeit?**

☐ Stunden

**56** **Wie viel Berufserfahrung haben Sie ...**

☞ *Bitte ziehen Sie mögliche Zeiten einer längeren Arbeitsunterbrechung (z.B. Elternzeit) ab.*

**... im Berufsfeld der frühkindlichen Bildung, Betreuung und Erziehung insgesamt?**

☐ Jahre

**... in Ihrer derzeitigen Einrichtung?**

☐ Jahre

**57** **Wie viele Jahre haben Sie Leitungserfahrung?**

☞ *Bitte ziehen Sie mögliche Zeiten einer längeren Arbeitsunterbrechung (z.B. Elternzeit) ab.*

☐ Jahre

**58** **Wie hoch ist Ihr Brutto-Monatsgehalt?**

*Gemeint ist Ihr monatliches Gehalt, vor Abzug aller Steuern und Abgaben.*

☐ Euro pro Monat

Diese Frage möchte ich nicht beantworten 1 ☐

**59** **Erhalten Sie Sonder- bzw. Einmalzahlungen (z.B. Weihnachts- bzw. Urlaubsgeld)?**

Ja 1 ☐

Nein 2 ☐

**60** **Wie zufrieden sind Sie gegenwärtig mit Ihrer Arbeit?**

| Ganz und gar unzufrieden 0 | 1 | 2 | 3 | 4 | 5 | 6 | 7 | 8 | 9 | Ganz und gar zufrieden 10 |
|---|---|---|---|---|---|---|---|---|---|---|
| ☐ | ☐ | ☐ | ☐ | ☐ | ☐ | ☐ | ☐ | ☐ | ☐ | ☐ |

**61** **Geben Sie bitte für jeden der folgenden Bereiche an, inwieweit Sie persönlich gegenwärtig Bedarf an Fort- und Weiterbildung haben.**

*Bitte machen Sie in jeder Zeile eine Angabe.*

| **Bedarf an Fort- und Weiterbildung zum Thema...** | **Kein Bedarf 1** | **2** | **3** | **4** | **5** | **Sehr hoher Bedarf 6** |
|---|---|---|---|---|---|---|
| Zusammenarbeit mit Familien/Erziehungspartnerschaft | ☐ | ☐ | ☐ | ☐ | ☐ | ☐ |
| Spezifisches pädagogisches Konzept (z.B. Montessori, Fröbel) | ☐ | ☐ | ☐ | ☐ | ☐ | ☐ |
| Spezifisches pädagogisches Thema (z.B. Literacy/ Sprache, Mathematik, Naturwissenschaft) | ☐ | ☐ | ☐ | ☐ | ☐ | ☐ |
| Kinderschutz | ☐ | ☐ | ☐ | ☐ | ☐ | ☐ |
| Qualitätsentwicklung und -sicherung | ☐ | ☐ | ☐ | ☐ | ☐ | ☐ |
| Selbstmanagement | ☐ | ☐ | ☐ | ☐ | ☐ | ☐ |
| Arbeitsorganisation | ☐ | ☐ | ☐ | ☐ | ☐ | ☐ |
| Verwaltung | ☐ | ☐ | ☐ | ☐ | ☐ | ☐ |
| Finanzmanagement | ☐ | ☐ | ☐ | ☐ | ☐ | ☐ |
| IT-Nutzung | ☐ | ☐ | ☐ | ☐ | ☐ | ☐ |
| Teamleitung/-entwicklung | ☐ | ☐ | ☐ | ☐ | ☐ | ☐ |
| Konfliktmoderation im Team | ☐ | ☐ | ☐ | ☐ | ☐ | ☐ |
| Personalführung (z.B. Methoden für ein nützliches Feedback) | ☐ | ☐ | ☐ | ☐ | ☐ | ☐ |
| Praxisanleitung | ☐ | ☐ | ☐ | ☐ | ☐ | ☐ |
| Sonstiges, und zwar | ☐ | ☐ | ☐ | ☐ | ☐ | ☐ |

*Bitte angeben:*

☐

**62** **Haben Sie in den letzten 12 Monaten an einer Fort- und Weiterbildung teilgenommen?**

Ja 1 ☐ → ***Bitte weiter mit Frage 64***

Nein 2 ☐ → ***Bitte weiter mit Frage 63***

## 63 Aus welchen Gründen haben Sie in den letzten 12 Monaten nicht an Fort- und Weiterbildungen teilgenommen?

*Bitte machen Sie in jeder Zeile eine Angabe.*

| | 1 Ja | 2 Nein |
|---|---|---|
| Keine Zeit aufgrund von Personalmangel in der Einrichtung | ☐ | ☐ |
| Zu hohe Kosten | ☐ | ☐ |
| Familiäre/private Gründe | ☐ | ☐ |
| Keine passenden Fort- und Weiterbildungsangebote gefunden | ☐ | ☐ |
| Keine Freistellung vom Arbeitgeber erhalten | ☐ | ☐ |
| Kein Bedarf an Fort- und Weiterbildungen teilzunehmen | ☐ | ☐ |

→ ***Bitte weiter mit Frage 66***

## 64 An welchen Fort- und Weiterbildungen haben Sie in den letzten 12 Monaten teilgenommen? Bitte geben Sie das Thema und den Umfang an.

*Bitte machen Sie in jeder Zeile eine Angabe. Für alle Maßnahmen, die Sie angegeben haben, tragen Sie bitte auch deren Umfang in Tagen ein. Bitte runden Sie halbe Tage zu ganzen auf.*

| | 2 Nein | 1 Ja | | Umfang in Tagen |
|---|---|---|---|---|
| Zusammenarbeit mit Familien/Erziehungspartnerschaft | ☐ | ☐ | → | |
| Spezifisches pädagogisches Konzept (z.B. Montessori, Fröbel) | ☐ | ☐ | → | |
| Spezifisches pädagogisches Thema (z.B. Literacy/Sprache, Mathematik, Naturwissenschaft) | ☐ | ☐ | → | |
| Kinderschutz | ☐ | ☐ | → | |
| Qualitätsentwicklung und -sicherung | ☐ | ☐ | → | |
| Selbstmanagement | ☐ | ☐ | → | |
| Arbeitsorganisation | ☐ | ☐ | → | |
| Verwaltung | ☐ | ☐ | → | |
| Finanzmanagement | ☐ | ☐ | → | |
| IT-Nutzung | ☐ | ☐ | → | |
| Teamleitung/-entwicklung | ☐ | ☐ | → | |
| Konfliktmoderation im Team | ☐ | ☐ | → | |
| Personalführung (z.B. Methoden für ein nützliches Feedback) | ☐ | ☐ | → | |
| Praxisanleitung | ☐ | ☐ | → | |
| Sonstiges, und zwar | ☐ | ☐ | → | |

*Bitte angeben:*

## 65 Wurden die Kosten für Ihre letzte Fort- und Weiterbildung übernommen bzw. erstattet?

☞ *Bitte machen Sie nur eine Angabe.*

- Die Kosten wurden komplett übernommen bzw. erstattet 1 ☐
- Die Kosten wurden teilweise übernommen bzw. erstattet 2 ☐
- Die Kosten wurden nicht übernommen oder erstattet 3 ☐

## 66 Haben Sie eine Weiterbildung absolviert, die Sie speziell für Ihre Leitungstätigkeit qualifiziert?

- Ja 1 ☐ → **Und wann haben Sie die (letzte) Weiterbildung hierzu absolviert?**
  - Innerhalb der letzten 12 Monate 1 ☐
  - Vor mehr als 12 Monaten 2 ☐
- Nein 2 ☐

## 67 Wie oft kam es in den letzten 6 Monaten vor, dass Sie sich mit anderen Leitungen ausgetauscht haben?

| Nie 1 | 2 | 3 | 4 | 5 | Sehr häufig 6 |
|---|---|---|---|---|---|
| ☐ | ☐ | ☐ | ☐ | ☐ | ☐ |

## 68 Welche der folgenden Angebote macht Ihnen Ihr Träger, um Sie bei Ihren Leitungsaufgaben zu unterstützen?

☞ *Bitte machen Sie in jeder Zeile eine Angabe.*

| | 1 Ja | 2 Nein |
|---|---|---|
| Regelmäßige Feedback-Gespräche zur Leitungstätigkeit | ☐ | ☐ |
| Fort- und Weiterbildung für Leitungsaufgaben | ☐ | ☐ |
| Leitungstreffen (kollegiale Beratung) | ☐ | ☐ |
| Fachberatung | ☐ | ☐ |
| Supervision/Coaching | ☐ | ☐ |
| Teamentwicklungsmaßnahmen | ☐ | ☐ |
| Hospitation in anderen Einrichtungen | ☐ | ☐ |
| Verwaltungskraft | ☐ | ☐ |

## 69 Wie häufig finden persönliche Besprechungen zwischen Einrichtungsleitung und Träger statt?

- Nie 1 ☐
- Seltener als einmal im Jahr 2 ☐
- Einmal im Jahr 3 ☐
- Mehrmals im Jahr 4 ☐



7135/2020

**70** **Einrichtungsleitungen können auf verschiedene Weise in Entscheidungen des Trägers eingebunden werden. Im Folgenden werden verschiedene Aufgaben genannt, die sowohl den Bereich der Träger wie auch den der Leitungen betreffen. Bitte geben Sie an, in welcher Weise Sie am Entscheidungsprozess beteiligt werden.**
**In welcher Weise werden Sie als Einrichtungsleitung bei Entscheidungen, die folgende Trägeraufgaben betreffen, beteiligt?**

☞ *Bitte machen Sie in jeder Zeile eine Angabe.*

| | Nicht informiert 1 | Informiert 2 | Angehört 3 | Ideen aufgegriffen 4 | Mitbestimmung 5 | Vetorecht 6 | Alleinige Entscheidung der Leitung 7 |
|---|---|---|---|---|---|---|---|
| Einrichtungskonzeption | ☐ | ☐ | ☐ | ☐ | ☐ | ☐ | ☐ |
| Sprachförderkonzept | ☐ | ☐ | ☐ | ☐ | ☐ | ☐ | ☐ |
| Verfahren der Beobachtung und Dokumentation | ☐ | ☐ | ☐ | ☐ | ☐ | ☐ | ☐ |
| Gesundheitsförderung (z.B. gesunde Ernährung, Bewegungsförderung) | ☐ | ☐ | ☐ | ☐ | ☐ | ☐ | ☐ |
| Raum und Ausstattung | ☐ | ☐ | ☐ | ☐ | ☐ | ☐ | ☐ |
| Beteiligung an Bundes-/ Landesprogrammen | ☐ | ☐ | ☐ | ☐ | ☐ | ☐ | ☐ |
| Budgetverwaltung der Einrichtungen | ☐ | ☐ | ☐ | ☐ | ☐ | ☐ | ☐ |
| Einstellung von pädagogischem Personal | ☐ | ☐ | ☐ | ☐ | ☐ | ☐ | ☐ |
| Einarbeitungskonzept für neue MitarbeiterInnen | ☐ | ☐ | ☐ | ☐ | ☐ | ☐ | ☐ |
| Fort- und Weiterbildungen von MitarbeiterInnen | ☐ | ☐ | ☐ | ☐ | ☐ | ☐ | ☐ |
| Maßnahmen zur Gesundheitsförderung der MitarbeiterInnen | ☐ | ☐ | ☐ | ☐ | ☐ | ☐ | ☐ |
| Öffnungszeiten | ☐ | ☐ | ☐ | ☐ | ☐ | ☐ | ☐ |
| Kriterien für die Platzvergabe | ☐ | ☐ | ☐ | ☐ | ☐ | ☐ | ☐ |
| Durchführung der Platzvergabe | ☐ | ☐ | ☐ | ☐ | ☐ | ☐ | ☐ |

## 71 Wie wahrscheinlich ist es, dass Sie in den nächsten 12 Monaten ...

☞ *Bitte machen Sie in jeder Zeile eine Angabe.*

| | Sehr unwahr-scheinlich 1 | 2 | 3 | 4 | 5 | Sehr wahr-scheinlich 6 |
|---|---|---|---|---|---|---|
| ... sich um eine Stelle in einer nächsthöheren Position bemühen? | ☐ | ☐ | ☐ | ☐ | ☐ | ☐ |
| ... ein fachrelevantes Studium aufnehmen? | ☐ | ☐ | ☐ | ☐ | ☐ | ☐ |
| ... sich eine andere Tätigkeit im Arbeitsfeld der Kinder- und Jugendhilfe suchen? | ☐ | ☐ | ☐ | ☐ | ☐ | ☐ |
| ... sich im Bereich der Frühpädagogik selbständig machen? | ☐ | ☐ | ☐ | ☐ | ☐ | ☐ |
| ... sich eine Arbeit in einem anderen Berufsfeld suchen? | ☐ | ☐ | ☐ | ☐ | ☐ | ☐ |
| ... in eine andere Stadt/Gegend ziehen, in der Sie bessere Arbeitsbedingungen vorfinden? | ☐ | ☐ | ☐ | ☐ | ☐ | ☐ |
| ... Ihre Arbeit in dieser Einrichtung kündigen oder sich in eine andere Einrichtung versetzen lassen? | ☐ | ☐ | ☐ | ☐ | ☐ | ☐ |
| ... Ihre Arbeit bei diesem Träger kündigen? | ☐ | ☐ | ☐ | ☐ | ☐ | ☐ |

## 72 Inwieweit beeinträchtigen die folgenden Aspekte die pädagogische Arbeit in Ihrer Kindertageseinrichtung?

☞ *Bitte machen Sie in jeder Zeile eine Angabe.*

| | Keine Beein-trächtigung 1 | 2 | 3 | 4 | 5 | Sehr starke Beein-trächtigung 6 |
|---|---|---|---|---|---|---|
| Unzureichende finanzielle und sachliche Ausstattung der Kindertageseinrichtung | ☐ | ☐ | ☐ | ☐ | ☐ | ☐ |
| Behördliche Vorschriften | ☐ | ☐ | ☐ | ☐ | ☐ | ☐ |
| Personalausfälle | ☐ | ☐ | ☐ | ☐ | ☐ | ☐ |
| Mangel an pädagogischen Fachkräften | ☐ | ☐ | ☐ | ☐ | ☐ | ☐ |
| Mangelndes Engagement und Unterstützung durch die Eltern bzw. Erziehungsberechtigten | ☐ | ☐ | ☐ | ☐ | ☐ | ☐ |
| Mangelnde Möglichkeiten und Unterstützung für meine eigene Fort- und Weiterbildung | ☐ | ☐ | ☐ | ☐ | ☐ | ☐ |
| Mangelnde Möglichkeiten und Unterstützung für die Fort- und Weiterbildung der pädagogischen Fachkräfte | ☐ | ☐ | ☐ | ☐ | ☐ | ☐ |
| Mangelnde Unterstützung durch den Träger | ☐ | ☐ | ☐ | ☐ | ☐ | ☐ |
| Mangelnde Unterstützung durch die Fachberatung | ☐ | ☐ | ☐ | ☐ | ☐ | ☐ |

## 73 Kommen wir nun zu den Räumen Ihrer Kindertageseinrichtung:

☞ *Bitte zählen Sie jeden Raum nur einmal.*

**Aus welchen und wie vielen Räumen besteht die Einrichtung?**

☞ *Bitte machen Sie in jeder Zeile eine Angabe.*

| | Nein [2] | Ja [1] | | Anzahl der Räume |
|---|---|---|---|---|
| Gruppen- und ergänzende Nebenräume für die pädagogische Arbeit mit den Kindern | ☐ | ☐ | → | ☐ |
| Schlafräume (ausschließlich dafür) | ☐ | ☐ | → | ☐ |
| Weitere Räume für die Kinder (z.B. Bastelraum, Bewegungsraum) | ☐ | ☐ | → | ☐ |
| Personalräume | ☐ | ☐ | → | ☐ |
| Sonstige Räume (z.B. Küche, Sanitärräume, Flur) | ☐ | ☐ | → | ☐ |

## 74 Bitte geben Sie für den Innenbereich Ihrer Einrichtung die Gesamtgröße der Räumlichkeiten an.

☞ *Wenn Sie die Gesamtgröße des Innenbereichs nicht kennen, schätzen Sie bitte so genau Sie können. Bitte auf ganze Zahlen runden.*

☐ qm

## 75 Verfügt Ihre Kindertageseinrichtung über ein Außengelände?

Ja 1 ☐ → ***Bitte weiter mit Frage 76***

Nein 2 ☐ → ***Bitte weiter mit Frage 77***

## 76 Bitte geben Sie die Gesamtgröße des Außengeländes Ihrer Einrichtung an.

☞ *Wenn Sie die Gesamtgröße des Außengeländes nicht kennen, schätzen Sie bitte so gut Sie können. Bitte auf ganze Zahlen runden.*

☐ qm

## 77 Gibt es in Ihrer Kindertageseinrichtung Standards für die Verpflegung (z.B. DGE-Qualitätsstandards, Bremer Checkliste)?

Ja 1 ☐

Nein 2 ☐

## 78 Welche Arten von Verpflegung bieten Sie an?

☞ *Bitte machen Sie in jeder Zeile eine Angabe.*

| | Ja [1] | Nein [2] |
|---|---|---|
| Frühstück | ☐ | ☐ |
| Mittagessen | ☐ | ☐ |
| Zwischenmahlzeiten | ☐ | ☐ |

(Noch) gar kein Verpflegungsangebot ☐

## 79 Wie viele Kinder nehmen in Ihrer Kindertageseinrichtung an der Mittagsverpflegung teil?

☐ Anzahl der Kinder, die an der Mittagsverpflegung teilnehmen

## 80 Fallen neben den regulären Gebühren Zusatzkosten für die Eltern an? Und wenn ja, in welcher Höhe?

☞ *Mit regulären Gebühren sind hier die für die Familien entstehenden Kosten für die in Anspruch genommenen Betreuungsstunden des Kindes gemeint ohne Verpflegungskosten, wie z.B. das Mittagessen. Bitte machen Sie in jeder Zeile eine Angabe.*
*Für alle Maßnahmen, die Sie angegeben haben, tragen Sie bitte auch die Höhe der Kosten in Euro ein.*

| | Nein [2] | Ja [1] | | Höhe der Kosten in Euro |
|---|---|---|---|---|
| Kosten für die Verpflegung | ☐ | ☐ | → | |
| Bastelgeld | ☐ | ☐ | → | |
| Optionale Angebote (z.B. musikalische Früherziehung) | ☐ | ☐ | → | |
| Kopiergeld | ☐ | ☐ | → | |
| Kosten für Mitgliedschaft (z.B. Förderverein) | ☐ | ☐ | → | |
| Sonstiges | ☐ | ☐ | → | |

## 81 Gab es in den letzten 12 Monaten in Ihrer Einrichtung hinsichtlich der Zusammenarbeit mit Eltern und Familien folgende Angebote?

☞ *Bitte machen Sie in jeder Zeile eine Angabe.*

| | Ja [1] | Nein [2] |
|---|---|---|
| Entwicklungsgespräche | ☐ | ☐ |
| Individuelle Beratungsangebote, z.B. zu Fragen der Erziehung, Bildung und Betreuung | ☐ | ☐ |
| Vermittlung von Fachärzten, Förderangeboten oder therapeutischen Angeboten | ☐ | ☐ |
| Vermittlung von Kontakten zu sozialen Diensten für Eltern und Familien (z.B. Gesundheits-, Ehe- oder Erziehungsberatung; Dienste, die Familien mit Migrationshintergrund bei der Integration unterstützen und beraten) | ☐ | ☐ |
| Mitbestimmungsmöglichkeiten, wie z.B. Entscheidung über Essensauswahl oder Ausflüge | ☐ | ☐ |
| Beteiligung an der Gestaltung des pädagogischen Alltags | ☐ | ☐ |
| Elternabende | ☐ | ☐ |
| Elternbriefe | ☐ | ☐ |
| Veranstaltungen und Vorträge zu pädagogischen Themen (z.B. Medienerziehung) | ☐ | ☐ |
| Veranstaltungen mit Eltern und Familien (z.B. Feste, Ausflüge, Projekte) | ☐ | ☐ |
| Elterntreffs | ☐ | ☐ |
| Mitbestimmungsgremien, wie z.B. Elternbeirat | ☐ | ☐ |
| Elternbefragung | ☐ | ☐ |
| Elternkurse, wie z.B. Sprachkurse, Kochkurse oder Nähkurse | ☐ | ☐ |
| Hospitation der Eltern in der Kita (außerhalb der Eingewöhnung) | ☐ | ☐ |
| Dokumentation des pädagogischen Alltags auf Wochenplänen oder Schautafeln | ☐ | ☐ |
| Besuche der pädagogischen Fachkräfte in den Familien | ☐ | ☐ |


7135/2020

## 82 Welche Angebote gibt es in Ihrer Einrichtung für eine kultursensible Zusammenarbeit mit Familien?

*Bitte machen Sie in jeder Zeile eine Angabe.*

| | 1 Ja | 2 Nein |
|---|---|---|
| Aushänge, Informationsblätter und Homepage sind in mehreren Sprachen verfasst | ☐ | ☐ |
| Personal, das sich mit (manchen) Kindern und Eltern in deren Herkunftssprache unterhalten kann | ☐ | ☐ |
| Es werden auch Festtage anderer Kulturen und Religionen gefeiert (neben Weihnachten z.B. auch Zuckerfest oder Opferfest) | ☐ | ☐ |
| Gelegenheiten für interkulturellen Austausch (Cafés, Vorträge) | ☐ | ☐ |
| Sonstiges | ☐ | ☐ |

## 83 Mit welchen Institutionen kooperiert Ihre Kindertageseinrichtung?

*Bitte machen Sie in jeder Zeile eine Angabe.*
*Kooperation heißt einen regelmäßigen Austausch sowie eine Zusammenarbeit mindestens einmal jährlich.*

| | Keine Kooperation 1 | Sporadische Kooperation 2 | Intensive Kooperation 3 |
|---|---|---|---|
| Grundschulen | ☐ | ☐ | ☐ |
| Vereine (z.B. Sportverein) | ☐ | ☐ | ☐ |
| Frühförderstellen | ☐ | ☐ | ☐ |
| Kirche | ☐ | ☐ | ☐ |
| Kulturelle Einrichtungen (z.B. Museen, Bibliotheken) | ☐ | ☐ | ☐ |
| Seniorenheime | ☐ | ☐ | ☐ |
| Betriebe | ☐ | ☐ | ☐ |
| Mütter- bzw. Familienzentren | ☐ | ☐ | ☐ |
| Volkshochschule | ☐ | ☐ | ☐ |
| Polizei, Feuerwehr | ☐ | ☐ | ☐ |
| Soziale Dienste für die Eltern (z.B. Gesundheits- oder Erziehungsberatung) | ☐ | ☐ | ☐ |
| Arztpraxen, ÄrztInnen | ☐ | ☐ | ☐ |
| Einrichtungen für Frühe Hilfen, Erziehungs- und Familienberatungsstelle | ☐ | ☐ | ☐ |

## 84 Durch welche Maßnahmen unterstützt Ihre Einrichtung den Übergang vom Kindergarten in die Grundschule?

*Bitte machen Sie in jeder Zeile eine Angabe.*

| | 1 Ja | 2 Nein |
|---|---|---|
| Austausch mit Grundschulen über Kinder, die in die Schule kommen sollen | ☐ | ☐ |
| Besuch von gemeinsamen Fortbildungen für pädagogische Fachkräfte und Grundschullehrkräfte | ☐ | ☐ |
| Bereitstellung von Entwicklungsberichten für Grundschulen | ☐ | ☐ |
| Besuch von Grundschullehrkräften in Ihrer Einrichtung zum Kennenlernen des pädagogischen Alltags | ☐ | ☐ |
| Organisation gegenseitiger Besuche der Kinder in Grundschulen und der Kindertageseinrichtung | ☐ | ☐ |
| Treffen mit Grundschullehrkräften (z.B. um einen gelungenen Übergang zu gestalten) | ☐ | ☐ |
| Aktivitäten für Eltern bzw. Erziehungsberechtigte zum Verständnis vom Übergang in die Grundschule | ☐ | ☐ |
| Bildungsangebote/Programme für Vorschulkinder | ☐ | ☐ |

## 85 Welches Geschlecht haben Sie?

☞ *Bitte machen Sie nur eine Angabe.*

- Männlich 1 ☐
- Weiblich 2 ☐
- Divers 3 ☐

## 86 In welchem Jahr sind Sie geboren?

☐ Geburtsjahr

## 87 In welchem Land sind Sie geboren?

- Deutschland (BRD und DDR) 1 ☐ → ***Bitte weiter mit Frage 89***
- Ein anderes Land, und zwar 2 ☐ → ***Bitte weiter mit Frage 88***

☞ *Bitte angeben: Bei politischen Veränderungen im Laufe der Jahre, geben Sie bitte die heutige, gegebenenfalls deutsche Bezeichnung an.*

☐

## 88 Seit wann leben Sie in Deutschland?

☞ *Falls Sie mehrmals nach Deutschland zugezogen sind, beziehen Sie sich bitte auf den letzten Zuzug!*

☐ Jahr

Seit meiner Geburt ☐

## 89 Haben Sie die deutsche Staatsangehörigkeit?

- Ja 1 ☐ → ***Bitte weiter mit Frage 90***
- Nein 2 ☐ → ***Bitte weiter mit Frage 91***

## 90 Wie haben Sie die deutsche Staatsangehörigkeit erworben?

☞ *Bitte machen Sie nur eine Angabe.*

- Durch Geburt 1 ☐
- Weil mindestens einer meiner Eltern die deutsche Staatsangehörigkeit hat 2 ☐
- Durch Heirat, Adoption, Einbürgerung oder als AussiedlerIn, und zwar 3 ☐

☞ *Bitte angeben:*

im Jahr ☐

## 91 In welchem Land liegt der Geburtsort Ihrer Mutter heute?

- Deutschland ☐
- Ein anderes Land, und zwar ☐

☞ *Bitte angeben: Bei politischen Veränderungen im Laufe der Jahre, geben Sie bitte die heutige, gegebenenfalls deutsche Bezeichnung an.*

☐

## 92 In welchem Land liegt der Geburtsort Ihres Vaters heute?

- Deutschland ☐
- Ein anderes Land, und zwar ☐

☞ *Bitte angeben: Bei politischen Veränderungen im Laufe der Jahre, geben Sie bitte die heutige, gegebenenfalls deutsche Bezeichnung an.*

☐

## 93 Welchen höchsten allgemeinbildenden Schulabschluss haben Sie?

☞ *Bitte machen Sie nur eine Angabe.*

| | | |
|---|---|---|
| SchülerIn, besuche eine allgemeinbildende Vollzeitschule | 1 | ☐ |
| Von der Schule abgegangen ohne Schulabschluss | 2 | ☐ |
| Hauptschulabschluss (Volksschulabschluss) oder gleichwertiger Abschluss | 3 | ☐ |
| Polytechnische Oberschule der DDR mit Abschluss der 8. oder 9. Klasse | 4 | ☐ |
| Realschulabschluss (Mittlere Reife) oder gleichwertiger Abschluss | 5 | ☐ |
| Polytechnische Oberschule der DDR mit Abschluss der 10. Klasse | 6 | ☐ |
| Fachhochschulreife | 7 | ☐ |
| Abitur/Allgemeine oder fachgebundene Hochschulreife (Gymnasium bzw. EOS, auch EOS mit Lehre) | 8 | ☐ |
| Einen anderen Schulabschluss, und zwar | 9 | ☐ |

☞ *Bitte angeben:*

| | | |
|---|---|---|
| Einen ausländischen Schulabschluss | 10 | ☐ |

☞ *Bitte Dauer in Jahren des ausländischen Schulabschlusses angeben*

## 94 Welches ist Ihr höchster beruflicher Ausbildungsabschluss?

☞ *Bitte machen Sie nur eine Angabe.*

| | | |
|---|---|---|
| SozialpädagogIn, SozialarbeiterIn (Dipl., Mag., MA) | 1 | ☐ |
| SozialpädagogIn, SozialarbeiterIn (BA) | 2 | ☐ |
| PädagogIn, ErziehungswissenschaftlerIn (Dipl., Mag., MA) | 3 | ☐ |
| PädagogIn, ErziehungswissenschaftlerIn (BA) | 4 | ☐ |
| HeilpädagogIn (Dipl., Mag., MA) | 5 | ☐ |
| HeilpädagogIn (BA) | 6 | ☐ |
| Staatlich anerkannte KindheitspädagogIn (MA) | 7 | ☐ |
| Staatlich anerkannte KindheitspädagogIn (BA) | 8 | ☐ |
| ErzieherIn | 9 | ☐ |
| HeilpädagogIn (Fachschule), HeilerzieherIn, HeilerziehungspflegerIn | 10 | ☐ |
| KinderpflegerIn | 11 | ☐ |
| FamilienpflegerIn, AssistentIn im Sozialwesen, soziale und medizinische HelferInnenberufe | 12 | ☐ |
| Sonstige soziale/sozialpädagogische Kurzausbildung, und zwar | 13 | ☐ |

☞ *Bitte angeben:*

| | | |
|---|---|---|
| Gesundheitsdienstberufe | 14 | ☐ |
| Verwaltungs-/Büroberufe | 15 | ☐ |
| Sonstiger Berufsausbildungsabschluss | 16 | ☐ |

☞ *Bitte angeben:*

| | | |
|---|---|---|
| PraktikantIn im Anerkennungsjahr | 17 | ☐ |
| Noch in Berufsausbildung | 18 | ☐ |
| Ohne abgeschlossene Berufsausbildung | 19 | ☐ |

**95** **An welcher Art Hochschule/Einrichtung haben Sie diesen Abschluss (bzw. den höchsten dieser Abschlüsse) erreicht?**

☞ *Bitte machen Sie nur eine Angabe.*

- Berufsakademie 1 ☐
- Verwaltungsfachhochschule 2 ☐
- Fachhochschule (University of Applied Sciences, auch: Ingenieurschule oder andere nicht-universitäre Hochschule) 3 ☐
- Universität (wissenschaftliche Hochschule, auch: Kunsthochschule, Pädagogische Hochschule, Theologische Hochschule) 4 ☐

**96** **Waren beim Ausfüllen des Fragebogens weitere Personen anwesend?**

☞ *Bitte machen Sie in jeder Zeile eine Angabe.*

| | 1 **Ja** | 2 **Nein** |
|---|---|---|
| MitarbeiterInnen, KollegInnen | ☐ | ☐ |
| Vorgesetzte | ☐ | ☐ |
| Andere Person | ☐ | ☐ |

**97** **Welches Datum haben wir heute?**

☐ Tag ☐ Monat

**98** **Haben Sie den Fragebogen am Stück ausgefüllt?**

- Ja 1 ☐
- Nein mit einer/mehreren Unterbrechungen 2 ☐

## Herzlichen Dank für Ihre Mithilfe.

Bitte schicken Sie den ausgefüllten Fragebogen
im portofreien Umschlag an infas zurück.



7135/2020
