# Supplementary material for: Establishing multi-perspective instruments in early education during COVID-19: measuring the implementation of protective measures and the subjective level of information about pandemic-related regulations
Source: Meas Instrum Soc Sci. 2022 May 12;4(1):7. doi: 10.1186/s42409-022-00033-2 (PMC9096761; doi:10.1186/s42409-022-00033-2)
Supplement: Supplementary file 5 — Additional file 5. Question translations [file 42409_2022_33_MOESM5_ESM.docx]

Translations:

**CKS question 19:**

How well is the implementation of the following measures working in your institution? (answer scale from 1 = Very bad to 5 = Very well; Missing value = Does not apply)

1. Airing regularly
2. Disinfecting furniture, door handles or toys regularly
3. Daily temperature measurements on children
4. Daily temperature measurements on personnel
5. Regularly washing hands with children
6. Regularly washing hands among personnel
7. Regularly testing personnel on COVID-19
8. Parents are wearing face masks (including face shields) when in contact with the institution.
9. Personnel is wearing face masks in certain situations.
10. Routes in the institution are marked and used (e.g. for parents or children of different groups).
11. Employees maintain social distancing among themselves.
12. Fixed assignment personnel to group
13. Group separation indoors
14. Group separation outdoors
15. Employees maintain social distancing between themselves and children of their group.
16. Employees maintain social distancing between themselves and children of other groups.

**CKS question 20:**

To what degree is pedagogic staff wearing face masks? (answer scale from 0 = Never to 5 = Always)

The pedagogic staff is wearing face masks …

1. … in contact with parents.
2. … in contact with colleagues (e.g. in break rooms, meetings).
3. … in contact with third parties (e.g. delivery persons, therapists).
4. … when putting on diapers or accompanying to the toilet.
5. … during pedagogic group work.
6. … in contact with children when social distancing is not possible (e.g. when looking at books together).
7. … in other situations.
